# Bioinspired Intelligent Soft Robotics: From Multidisciplinary Integration to Next‐Generation Intelligence

**DOI:** 10.1002/advs.202506296

**Published:** 2025-06-29

**Authors:** Xiaopeng Wang, Ruilai Wei, Zhongming Chen, Hao Pang, Haotian Li, Yang Yang, Qilin Hua, Guozhen Shen

**Affiliations:** ^1^ School of Integrated Circuits and Electronics Beijing Institute of Technology Beijing 100081 China

**Keywords:** actuation, bioinspired, embodied intelligence, intelligent machines, soft robotics

## Abstract

Soft robotics, distinguished by intrinsic compliance, biomimetic adaptability, and safe human‐environment interaction, has emerged as a transformative paradigm in next‐generation intelligent systems. Biological systems, refined through evolutionary optimization, exhibit unparalleled multifunctionality in unstructured environments, inspiring the development of soft robots with energy‐efficient reconfiguration and environmental responsiveness. This review presents a comprehensive analysis of intelligent soft robotics via multidisciplinary integration, covering key aspects from bioinspired design principles to advanced functional implementation. Recent breakthroughs across four interconnected domains are systematically examined: 1) biomimetic actuation mechanisms that enhance actuation efficiency through innovative structural configurations; 2) programmable materials enabling adaptive morphology and tunable mechanical properties; 3) multiscale manufacturing techniques for fabricating complex heterogeneous structures; and 4) closed‐loop control strategies integrating artificial intelligence algorithms. While highlighting emerging applications in biomedical engineering, environmental exploration, and human‐machine interfaces, challenges such as actuation efficiency, material degradation, manufacturing limitations, nonlinear‐control complexity, and sensing instability under real‐world conditions are discussed. Furthermore, strategic research directions are identified to guide the development of next‐generation soft robots endowed with embodied intelligence and adaptive functionalities. Notably, by synergizing advances in materials science, mechanical engineering, and computational intelligence, soft robotics is poised to redefine the boundaries of intelligent machines across healthcare, exploration, and human augmentation.

## Graphical abstract

Soft robotics, featuring intrinsic compliance and biomimetic adaptability, emerges as transformative in next‐generation intelligent systems. This review outlines how advancements in four foundational domains—actuation, materials, manufacturing, and control—drive the evolution of bioinspired intelligent soft robotics, poised to redefine the boundaries of intelligent machines across healthcare, exploration, and human augmentation.

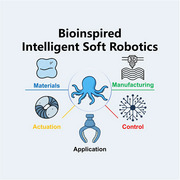

## 1 Introduction

Robotics are engineered to automate specific tasks and enhance human productivity through programmable systems capable of performing complex, hazardous, or repetitive operations.^[^ ^1^, ^2^ ^]^ Traditional rigid robots, which rely on kinematic pairs (rigid joints) with stiff components like metal or plastic, face challenges such as structural inflexibility, limited adaptability, and safety concerns in dynamic environments.^[^ ^3^, ^4^ ^]^ In contrast, soft robotics – inspired by the locomotive strategies of natural organisms such as octopuses,^[^ ^12^, ^13^, ^14^, ^15^, ^16^ ^]^ snailfish,^[^ ^17^, ^18^ ^]^ jellyfish,^[^ ^19^, ^20^ ^]^ worms,^[^ ^21^, ^22^, ^23^ ^]^ elephant trunks,^[^ ^24^ ^]^ dragonflies,^[^ ^25^ ^]^ and venus flytraps^[^ ^26^ ^]^ – offers solutions to these limitations through compliant, bioinspired designs.^[^ ^5^, ^6^, ^7^, ^8^, ^9^, ^10^, ^11^ ^]^ Biological systems have evolutionarily optimized multifunctional capabilities, including locomotion, sensing, and adaptation, with remarkable efficiency, robustness, and environmental responsiveness. These naturally engineered traits position living organisms as archetypes for developing intelligent, flexible, and resilient robotic systems. Consequently, biomimicry is not only a source of aesthetic or mechanical inspiration but also a fundamental design philosophy in soft robotics, guiding material selection, structural layout, and functional behavior.^[^ ^27^ ^]^ Soft robotics is designed from multidisciplinary integration of actuation, material, manufacturing, and control technologies with highly flexible and deformable materials, which work synergistically to enable the unique capabilities for adaptive movements and interactions. These advancements show great promises for applications in environmental exploration, medical interventions, wearable devices, and disaster response.

The actuation architecture and mechanisms significantly influence the mobility, flexibility, and adaptability of soft robots in complex environments, playing a decisive role in their overall performance.^[^ ^7^ ^]^ As highly flexible and compliant entities, actuation systems for soft robots must be flexible, stable, and efficient, which affects their performance in dynamic and evolving environments.^[^ ^28^, ^29^ ^]^ Soft robots encounter stability and control challenges in their actuation systems, limiting their accuracy and reliability. While early research primarily focused on traditional driving methods such as pneumatics,^[^ ^30^, ^31^, ^32^, ^33^ ^]^ hydraulics,^[^ ^34^, ^35^, ^36^ ^]^ to enhance flexibility and efficiency, they still suffered from low efficiency, imprecise control, and system complexity. Bioinspired actuation mechanisms—such as muscle‐like contractions, hydrostatic skeletons, and peristaltic motions—offer potential solutions by emulating the efficient, distributed, and adaptive movement strategies observed in animals like worms, fish, and mollusks. Future research will explore bioinspired actuation mechanisms based on intelligent materials—such as shape memory materials,^[^ ^67^, ^81^ ^]^ stimuli‐responsive polymers,^[^ ^63^ ^]^ and functional composites—as well as chemical reactions,^[^ ^56^, ^57^, ^62^ ^]^ to improve actuation efficiency, stability, and control accuracy, thereby further unleashing the potential of soft robots.

The actuation mechanisms of robotics are derived from the intrinsic responsiveness of materials to external stimuli. Therefore, the design and selection of materials play a critical role in determining the performance, stability, and long‐term reliability of soft robots. Key material properties such as flexibility, strength, durability, and adaptability (defined as a material's ability to maintain functional performance under varying external conditions such as temperature, humidity, or pressure) significantly impact the functionality of robots across various environments.^[^ ^7^ ^]^ Previous research mainly concentrated on developing novel flexible materials, such as shape‐memory materials^[^ ^68^, ^82^ ^]^ and hydrogels,^[^ ^70^, ^71^, ^72^, ^73^ ^]^ which respond to external stimuli like light,^[^ ^42^, ^43^ ^]^ electricity,^[^ ^47^, ^48^ ^]^ and heat,^[^ ^40^, ^41^ ^]^ showing progress in enhancing robotic motion performance and response speed. However, challenges such as limited durability, high control complexity, and low efficiency persist, constraining their practical applications in real‐world scenarios. Biological tissues offer a compelling reference in this regard: their hierarchical, hydrated, and stimuli‐responsive nature inspires the development of multifunctional materials with tunable mechanical properties and self‐healing capabilities, paving the way for more robust and adaptive soft robots. To address these technical challenges, future research is expected to emphasize the optimization of soft robotic materials, particular focusing on intelligent materials that autonomously adjust their properties in response to environmental changes, potentially offering more flexible, reliable, and efficient actuation solutions for soft robots and broadening their practical applications.

Manufacturing methods for soft robotics play a crucial role in determining their adaptability (defined as the ability of the robot to conform to diverse structural configurations and application scenarios enabled by modular or multi‐material fabrication), control accuracy and operational efficiency in real‐world environments.^[^ ^88^, ^89^, ^90^ ^]^ Despite the utilization of high‐performance materials and advanced actuation mechanisms, soft robots still face numerous challenges such as inadequate control precision, slow response speeds, and limited durability. To address these challenges, researchers are actively exploring novel flexible materials and advanced manufacturing systems to boost the performance of soft robots. Nevertheless, existing technologies face constraints like low manufacturing efficiency and high production costs. Many biological systems, such as spider webs, insect wings, or fish scales, form intricate and multifunctional structures through decentralized, energy‐efficient, and self‐organized growth processes—offering valuable inspiration for the development of scalable, bioinspired manufacturing strategies such as multimaterial 3D printing or soft lithography. To address these issues, recent efforts have focused on high‐resolution multi‐material 3D printing and improved soft lithography using reusable molds and low‐temperature‐curing elastomers. These approaches enhance integration, reduce curing time, and support complex structural fabrication. Future work should aim to standardize processing parameters such as layer adhesion, curing conditions, and feature resolution to improve reproducibility and scalability.^[^ ^64^ ^]^ By addressing these issues, soft robots could become more reliable, efficient, and cost‐effective, opening up broader opportunities for practical, real‐world applications. Breakthroughs in these areas, when integrated with intelligent control strategies, can elevate soft robotics to high‐dimensional levels of automation and intelligence.^[^ ^104^ ^]^ This advancement will enable broader applications and further promote technological innovation.

Control strategies of soft robotics directly influence their ability to perform tasks efficiently and accurately in complex and diverse environments, making them a focal point of research in this field. Compared to traditional rigid robots, controlling soft robots is significantly more intricate and challenging, especially concerning multi‐degree‐of‐freedom movements.^[^ ^104^ ^]^ Achieving precise localization, stable control and efficient coordination in such scenarios remains a major unsolved issue. Due to their highly flexible and deformable attributes, soft robots face even greater difficulties in maintaining the stability and predictability of their movements compared to traditional robots. This challenge becomes particularly when executing complex tasks or responding to unforeseen situations, where control accuracy and response speed are severely tested. Researchers have explored innovative modeling methods^[^ ^107^ ^]^ to dynamically adjust and optimize robot motion trajectories in real‐time within evolving environments, by developing more accurate mathematical models and integrating simulations with experimental approaches.^[^ ^108^ ^]^ In contrast, biological organisms—such as cephalopods, insects, or the human nervous system—demonstrate robust real‐time control via distributed neuromuscular architectures, reflex loops, and adaptive feedback mechanisms, offering a conceptual basis for neuromorphic and decentralized control in soft robots. However, despite some progress, challenges related to precise control, real‐time responsiveness, and adaptive regulation remain unsolved. Therefore, further breakthroughs in control algorithms,^[^ ^114^ ^]^ intelligent systems,^[^ ^115^ ^]^ and sensor fusions^[^ ^110^, ^111^, ^112^, ^113^ ^]^ are crucial for soft robots to achieve stable, efficient operation and enhanced adaptability in complex scenarios.

**Figure 1:**
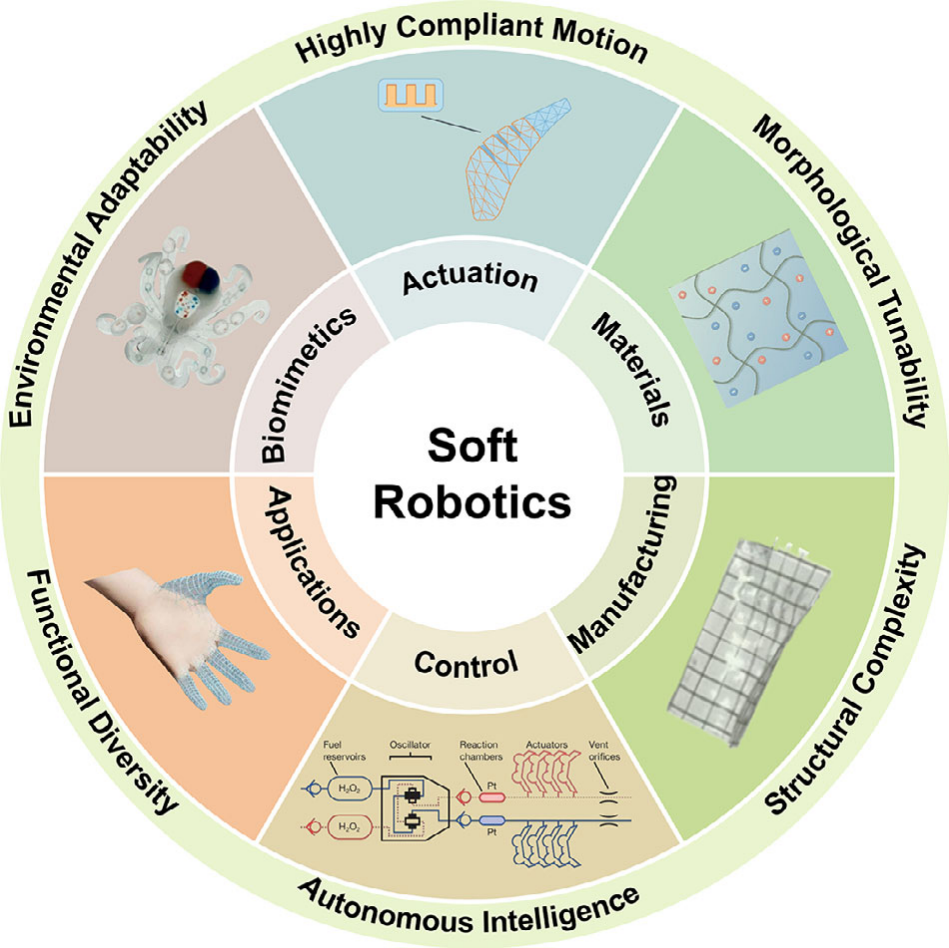
The organizational structure diagram of soft robotics, including bioinspired significance,^[^ ^12^ ^]^ actuation mechanisms, material designs,^[^ ^72^ ^]^ manufacturing methods,^[^ ^170^ ^]^ control strategies,^[^ ^12^ ^]^ and practical applications.^[^ ^11^ ^]^ Reproduced with permission.^[^ ^12^ ^]^ Copyright 2016, Springer Nature. Reproduced with permission.^[^ ^72^ ^]^ Copyright 2020, Elsevier. Reproduced with permission.^[^ ^170^ ^]^ Copyright 2015, John Wiley & Sons. Reproduced with permission.^[^ ^11^ ^]^ Copyright 2020, AAAS.

The development of soft robotics relies on interdisciplinary collaboration across materials science, mechanical engineering, control theory, computer science, and artificial intelligence. Progress in this field is driven by comprehensive innovations in material design, actuation systems, precise control, and intelligent decision‐making. This paper provides a comprehensive review of the latest advancements in soft robotics, covering biomimetics, actuation mechanisms, material selection, manufacturing methods, control strategies, and practical applications, as illustrated in **Figure** **1**. Additionally, this review highlights the future potential of soft robotics across healthcare, industry, and extreme environments. It examines current technical challenges and recent advancements in materials, actuation, sensing, manufacturing, and control while proposing future directions to promote intelligent functions and sustainable development. As the field advances, bioinspiration will continue to serve as a core design strategy. By emulating the adaptability, efficiency, and multifunctionality of natural organisms, soft robotics can evolve into more intelligent, resilient, and versatile systems suited for complex real‐world applications.

## 2 Bioinspired Implementation of Soft Robotics

Soft robotics, as an emerging technology, draws profound bionic inspiration from nature's flora and fauna. Organisms like octopuses, jellyfish, and climbing plants, exemplify exceptional traits such as agility, flexibility, and morphing abilities, enabling them to efficiently perceive, grasp, and thrive in intricate and ever‐changing environments. The unique capabilities serve as invaluable models for soft robot design, propelling innovation in advanced technologies.^[^ ^1^ ^]^

By emulating the supple structures of these organisms, soft robots demonstrate exceptional agility in traversing narrow and complex spaces, adeptly adapting to environmental changes, and ensuring operational safety. In particular, many biological structures exhibit motion strategies—such as pulsation, peristalsis, or undulatory propulsion—that naturally optimize energy transfer and force distribution, offering clear inspiration for improving actuation efficiency in soft robotic systems. Bioinspired exploration in soft robots not only expands the horizons of robotic applications but also enhances their adaptability and task execution efficiency in complex scenarios. The bioinspired essence of soft robotics lies in harmonizing nature's wisdom with modern technology, propelling robotics toward a more intelligent and flexible direction.^[^ ^5^ ^]^ Rather than classifying soft robots by individual species, this section reorganizes representative bioinspired designs into functional motion categories—including tentacle/fin‐based propulsion, peristaltic crawling, flapping flight, and rapid grasping—each supported by multiple case studies to illustrate shared actuation strategies and structural advantages.

### 2.1 Morphological Adaptation Inspired by Octopus Arms and Jellyfish Bodies

Soft organisms such as octopuses and jellyfish exhibit exceptional morphing capabilities and fluid‐environment adaptability, which have inspired a range of soft robots capable of large deformation, distributed actuation, and energy‐efficient propulsion. This section highlights representative designs that utilize compliant materials and asymmetric architectures to mimic these biological traits.

#### 2.1.1 Octopus‐Inspired Soft Robots

**Figure 2:**
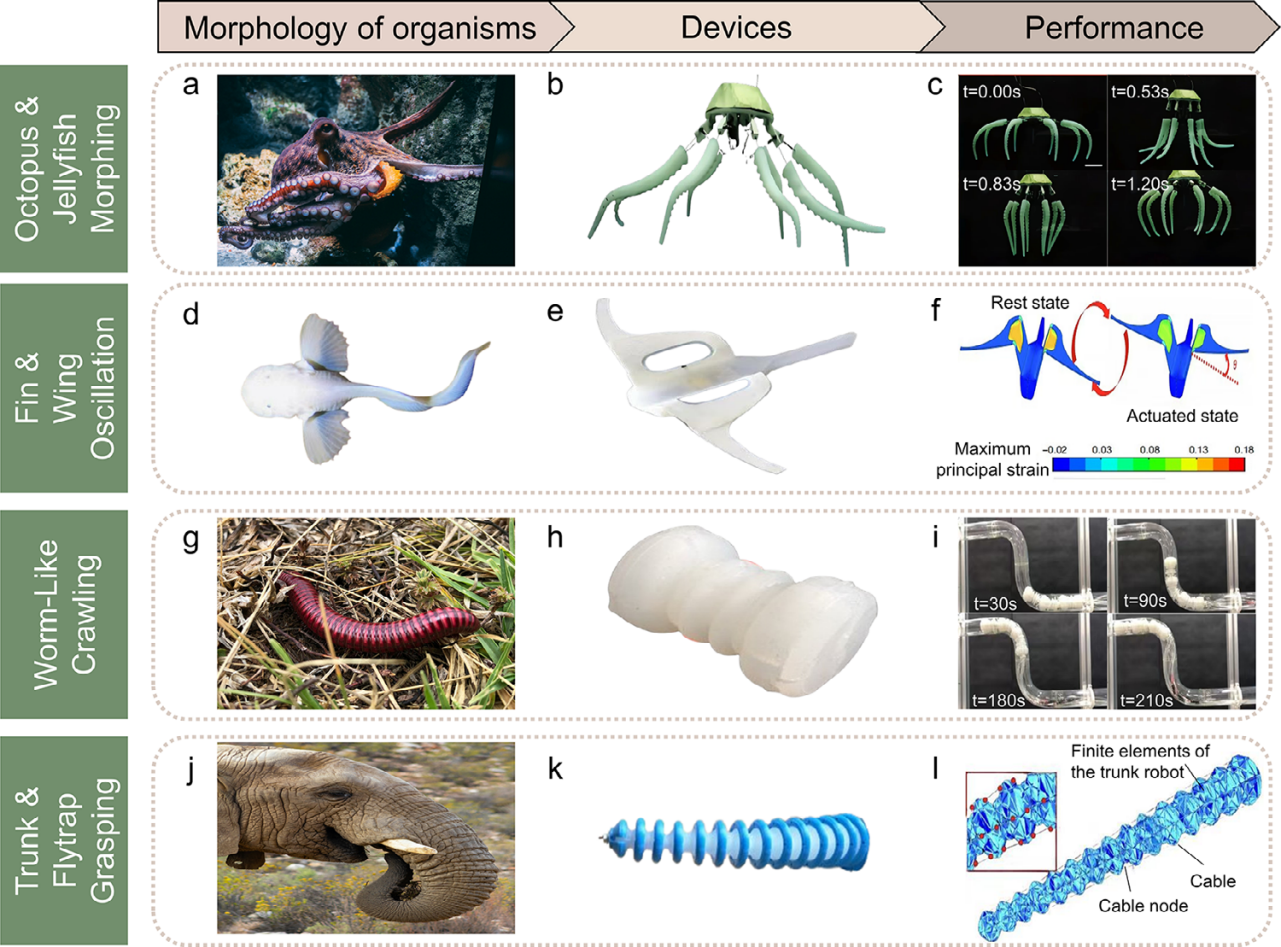
Representative bioinspired soft robots categorized by functional motion types. a) Image of an octopus, exhibiting flexible arms and decentralized muscular control. b) Soft robot mimicking the octopus morphology, featuring multiple flexible arms for multidirectional motion.^[^ ^15^ ^]^ c) Sequential snapshots of the robot's swimming motion, illustrating asymmetric actuation between power stroke and recovery stroke with a time ratio of 2:1.^[^ ^15^ ^]^ (b‐c) Reproduced with permission.^[^ ^15^ ^]^ Copyright 2025, MDPI. d) Image of the Mariana hadal snailfish, adapted to extreme deep‐sea pressure with soft, distributed body structure.^[^ ^18^ ^]^ Reproduced with permission.^[^ ^18^ ^]^ Copyright 2019, Springer Nature. e) Snailfish‐inspired soft robot designed for deep‐sea propulsion using dielectric elastomer actuation.^[^ ^17^ ^]^ f) Finite element analysis (FEA) of the robot's fin, showing a transition from resting to actuated state under voltage and corresponding principal strain distribution; fin oscillation is driven by angular deformation θ.^[^ ^17^ ^]^ (e‐f) Reproduced with permission.^[^ ^17^ ^]^ Copyright 2021, Springer Nature. g) Image of an annelid worm, exhibiting peristaltic crawling. h) Modular design of a worm‐inspired actuator, enabling bidirectional contraction for confined‐space locomotion.^[^ ^21^ ^]^ i) Time‐lapse sequence showing a worm‐inspired robot traversing an S‐shaped pipeline, powered by multiple serial pneumatic actuators.^[^ ^21^ ^]^ (h‐i) Reproduced with permission.^[^ ^21^ ^]^ Copyright 2021, John Wiley & Sons. j) Image of an elephant trunk, highlighting its high degrees of freedom and dexterous manipulation capability. k) Elephant trunk‐inspired soft robot implementing spatial bending and grasping functions.^[^ ^107^ ^]^ l) Finite element modeling of the trunk robot, where its geometry is discretized into cable‐linked mesh elements to simulate dynamic behavior and ensure precise control.^[^ ^107^ ^]^ (k‐l) Reproduced with permission.^[^ ^107^ ^]^ Copyright 2022, Elsevier.

The biological attribute of octopuses serves as crucial inspirations for the design of soft robots. Octopuses possess bodies primarily comprised of soft tissues, devoid of skeletal structures, granting them exceptional flexibility and deformability to adapt to diverse spaces or environments, as shown in **Figure** **2**a. Drawing from this feature, researchers have designed robots without skeletons to enhance flexibility in constrained spaces or intricate tasks. In 2016, Wehner et al. developed a fully soft robot called Octobot leveraging microfluidic logic for liquid flow regulation and fuel decomposition catalysis. Through the integration of soft materials and multi‐material 3D printing, Octobot achieved autonomous functionality without external control.^[^ ^12^ ^]^

In addition to its highly flexible and boneless body, the tentacles of the octopus exhibit remarkable flexibility and gripping prowess, each adorned with thousands of suction cups facilitating precise object control and manipulation. This feature also provides valuable inspiration for the design of soft robots, particularly for tasks requiring delicate operations or manipulation of small items, such as in minimally invasive surgery. For instance, Wu et al. designed a soft gripper, inspired by octopus tentacles featuring luminescent suction cups. This gripper proficiently senses and grasps objects of various sizes and shapes, showcasing robust gripping force and suction capabilities through 3D printing and modular casting techniques, making it adept at handling flat, irregular, dispersed, or moving objects and performing effectively in turbid water environments.^[^ ^13^ ^]^

Furthermore, the octopus tentacles exhibit an asymmetric stiffness gradient and fluidic internal distribution, which has been replicated in robotic systems to improve propulsion efficiency and reduce actuation energy loss. Zhang et al. designed a bioinspired octopus swimming robot (Figure 2b), featuring soft tentacles with asymmetric stiffness and a quick‐return mechanism, enabling the adjustment of the time ratio between recovery stroke and power stroke.^[^ ^14^ ^]^ Experimental results show that the tentacles with 70% incision closely resembled the movement of real octopus tentacles. Operating at a stroke time ratio of 2.0:1, the robot demonstrated excellent underwater swimming performance (Figure 2c).^[^ ^15^ ^]^ Such bioinspired structural configurations directly contribute to the enhanced actuation efficiency of soft actuators in aquatic environments.

#### 2.1.2 Jellyfish‐Inspired Soft Robots

Jellyfish, with their transparent, soft gelatinous bodies and efficient “pulsating” swimming motion, achieve low‐energy propulsion and effective camouflage in water, serving as a significant inspiration for soft robot design.^[^ ^19^ ^]^ However, replicating the appearance and functionality of jellyfish poses a technical challenge due to the absence of transparent actuators. Wang et al. designed a transparent soft robot inspired by the moon jellyfish, utilizing optimized dielectric elastomer actuators (DEAs), combined with AgNWs/PEDOT:PSS hybrid electrodes with a multilayer structure. These enhanced DEAs achieved 146% area strain and 89% light transmittance under high voltage, improving mechanical performance and durability. The rhythmic contraction and expansion motion, directly inspired by jellyfish bell deformation, enables a highly efficient propulsion method with minimal energy loss per cycle, offering a model for soft robots requiring sustained motion with low power input. This advancement enabled the robot to mimic the movement and camouflage capabilities of natural jellyfish,^[^ ^20^ ^]^ with applications ranging from underwater behavior studies to exploration and security monitoring, poised for further enhancement through self‐sensing and reinforcement learning.

### 2.2 Bioinspired Oscillatory Locomotion Based on Fish Fins and Insect Wings

Organisms such as snailfish and dragonflies rely on oscillating fins or flapping wings for efficient propulsion in aquatic or aerial environments. By replicating these rhythmic motions using dielectric elastomer actuators and soft appendages, soft robots can achieve agile, low‐power movement. This section introduces soft robotic systems inspired by these dynamic locomotion principles.

#### 2.2.1 Snailfish‐Inspired Soft Robots

The deep‐sea environment presents daunting challenges to electromechanical devices due to extreme water pressure, typically requiring rigid hulls and pressure compensation systems. However, deep‐sea organisms, like snailfish (Figure 2d) thrive sans such systems, relying on distributed skulls and flapping pectoral fins for survival, inspiring soft robot designs.^[^ ^17^, ^18^ ^]^ Li et al. developed a tetherless soft robot (Figure 2e) equipped with autonomous power, control, and actuation modules. This robot uses a silicone matrix to shield electronic components and employs dielectric elastomer (DE) muscles for propulsive fin flapping (Figure 2f). With a distributed electronic component layout, the robot underwent trials in the Mariana Trench and the South China Sea, showcasing its pressure resistance and highlighting the potential of lightweight, flexible devices for deep‐sea exploration.^[^ ^17^ ^]^ Notably, the bioinspired flapping fin configuration mimics the energy‐efficient propulsion mechanisms of snailfish, enabling the robot to generate forward thrust under high pressure while minimizing structural stress and energy consumption, thus enhancing actuation efficiency in extreme underwater environments.

#### 2.2.2 Dragonfly‐Inspired Soft Robots

Flapping soft robots draw inspiration from insects, birds, and aquatic creatures known for energy‐efficient and flexible control. Dragonflies, as a typical example, achieve efficient flight through the coordination of two pairs of wings. Their ability to independently adjust wing angles and twisting offers insights into developing flexible flapping structures. Chen et al. designed a bio‐inspired robot modeled after dragonflies, weighing 317 mg and propelled by two DEAs at a frequency of 350 Hz to power four wings. The design mimics dragonflies’ wing coordination to improve lift generation, and experimental results confirmed that in‐phase flapping led to higher lift efficiency compared to out‐of‐phase flapping. Experiments showed that under in‐phase flapping, the robot achieved a lift‐to‐weight ratio of 1.49, surpassing out‐of‐phase flapping by 19%. The interaction between the forewings and hindwings significantly affected lift and aerodynamic efficiency, laying the groundwork for future flight studies centered on feedback control.^[^ ^25^ ^]^

### 2.3 Peristaltic Crawling Mechanisms Inspired by Annelid Worms

Worm‐like organisms navigate complex environments through peristaltic motion driven by coordinated segmental contractions. Their locomotion mechanisms offer effective models for soft robots intended for pipeline inspection, confined space navigation, and adaptive crawling. This section focuses on pneumatic systems and modular architectures that replicate such biological strategies.

Industries rely on pipelines for transporting water, oil, and gas, necessitating robots for inspection, especially in hazardous environments. Unlike rigid robots that may damage surfaces, worms' soft bodies and diverse movements serve as an inspirational model. Worms (Figure 2g) navigate using wave‐like muscle contractions and relaxations, forming longitudinal undulations for crawling and displaying flexibility in turning, burrowing, or serpentine movement.^[^ ^21^, ^22^, ^23^ ^]^ Liu et al. designed a worm‐inspired soft robot powered by pneumatic actuators (Figure 2h). Featuring a modular design, this robot adapts to various pipeline dimensions and contours, excelling in wet, oily, and underwater conditions. By mimicking the segmented body and peristaltic motion of worms, the robot achieves directional propulsion through sequential inflation of multiple chambers, improving its actuation efficiency and movement stability in confined spaces. The soft pneumatic actuators enable it to carry loads up to 11 times its weight, navigating complex pipeline networks for inspection and navigation tasks, and adeptly traverse S‐shaped pipelines (Figure 2i) with multiple series‐connected actuators, demonstrating promising applications in pipeline and tunnel inspection, cleaning and maintenance.^[^ ^21^ ^]^

### 2.4 Reconfigurable Grasping and Manipulation Inspired by Elephant Trunks and Plant Structures

Natural systems like the elephant trunk and Venus flytrap demonstrate remarkable capabilities in grasping, rapid closure, and structural reconfiguration. These mechanisms have been emulated in soft robots designed for dexterous manipulation, adaptive grasping, and responsive actuation. This section reviews robotic implementations that translate these biological strategies into functional actuators.

#### 2.4.1 Trunk‐Inspired Soft Robots

The elephant trunk (Figure 2j) is coordinated by dozens of muscles and exhibits high flexibility, multi‐degree‐of‐freedom motion, and precise grasping offering insights for soft robot design. Based on these characteristics, soft robots can perform tasks flexibly in complex environments and expand applications such as object handling, environmental exploration, and precision operations. Wu et al. developed a soft robotic elephant trunk (Figure 2k) and proposed a gain‐scheduled closed‐loop control method based on a dynamic finite element model. Employing the finite element method (FEM), the robot's geometry was discretized into “mesh” units (Figure 2l) to construct a nonlinear dynamic system.^[^ ^107^ ^]^ The muscle‐bundled structure and variable stiffness patterns observed in elephant trunks inspired the design of compliant actuators with directional stiffness tuning, which directly enhances motion precision and energy transmission during grasping and lifting. Linearizing the system under varied operating conditions, controllers were tailored for each subspace to facilitate seamless control transitions. This approach enhances the flexibility and control performance of high‐dimensional soft robots, rendering them versatile for diverse tasks.^[^ ^24^ ^]^

#### 2.4.2 Venus Flytraps‐Inspired Soft Robots

In addition to animals, plant species like the Venus flytrap inspire soft robot designs with rapid response, flexibility, and energy efficiency. Wang et al. introduced a bio‐inspired leaf emulating the Venus flytrap, incorporating cavity and flow channel structures to overcome single deformation mode limitations in typical soft robots. Experiments showed the leaf's ability to bend and close within 2 s under 0.4 kPa air pressure.^[^ ^26^ ^]^ This design, inspired by the snap‐through bistable mechanics of the flytrap, enables large, rapid deformations with minimal actuation input—translating into highly efficient energy storage and release mechanisms for soft robotic grippers and actuators. It offers insights into designing bio‐inspired soft robots and flexible actuators with multidimensional deformation, with future research aimed at optimizing structure and sealing while integrating sensors to bolster system performance.

The incorporation of bioinspired design principles in soft robotics has played a pivotal role in advancing key technological domains, including materials, actuation, sensing, manufacturing, and control. Biological organisms offer structural and functional paradigms characterized by compliance, adaptability, and multifunctionality, which have inspired the development of novel soft materials with enhanced elasticity, self‐healing, and multi‐responsive capabilities. Similarly, bioinspired locomotion strategies have guided the creation of diverse actuation mechanisms capable of achieving complex, high‐degree‐of‐freedom motion. In sensing, biological sensory systems have motivated the integration of multimodal and bioinspired sensors that improve perception in unstructured environments. Furthermore, the replication of intricate biological architectures has driven innovations in additive manufacturing techniques, enabling high‐resolution, multi‐material fabrication. In the realm of control strategies, the inherent nonlinear dynamics of soft biological systems have catalyzed the adoption of AI‐based and neuromorphic control frameworks. Beyond their immediate functional benefits, bioinspired designs also hold long‐term significance by promoting energy‐efficient behaviors through passive adaptability and structural intelligence. By mimicking organisms that thrive in unpredictable and unstructured environments, such designs can enhance the resilience, self‐regulation, and terrain compliance of soft robots. These characteristics not only reduce the computational and actuation burden but also expand the applicability of soft robots in dynamic, real‐world scenarios, thereby supporting sustainable and autonomous operation in the future. Collectively, these advancements underscore the foundational role of bioinspiration in shaping the multidisciplinary evolution of intelligent soft robotic systems.

## 3 Actuation Mechanisms of Soft Robotics

Organisms rely on ATP‐driven biochemical processes and hydraulic mechanisms to regulate muscle contraction and relaxation, enabling them to perform complex activities such as grasping, running, flying, and swimming that are adapted to dynamic environments.^[^ ^1^ ^]^ Soft actuators emulate the motion principles of biological musculoskeletal systems by performing functional movements pneumatically or hydraulically. For example, certain flexible chambers expand under pressure, simulating the hydraulic mechanism of an octopus's muscles.^[^ ^12^ ^]^ Concurrently, DEAs generate expansion and contraction through electrical stimulation, imitating the movement of natural muscles and skeletal joints.^[^ ^17^ ^]^ Mimicking the way in which organisms translate simple movements into complex functions has driven research and development in soft robotics actuation mechanisms.

Selecting appropriate actuator structures and driving strategies is pivotal to achieving bio‐inspired motion in soft robots.^[^ ^7^, ^28^ ^]^ Actuator structure influences motion patterns, deformation, control precision, and response speed while driving strategy impacts motion realization and functional performance. The two aspects are interdependent: requiring structural design to align with driving characteristics. Optimization and integration of actuator structure with driving strategies directly affect the robot's flexibility, precision, speed, force, and application range. Properly aligning actuator structures with driving strategies is imperative for efficient control and multifunctional applications in soft robotics.^[^ ^10^, ^29^ ^]^

**Figure 3:**
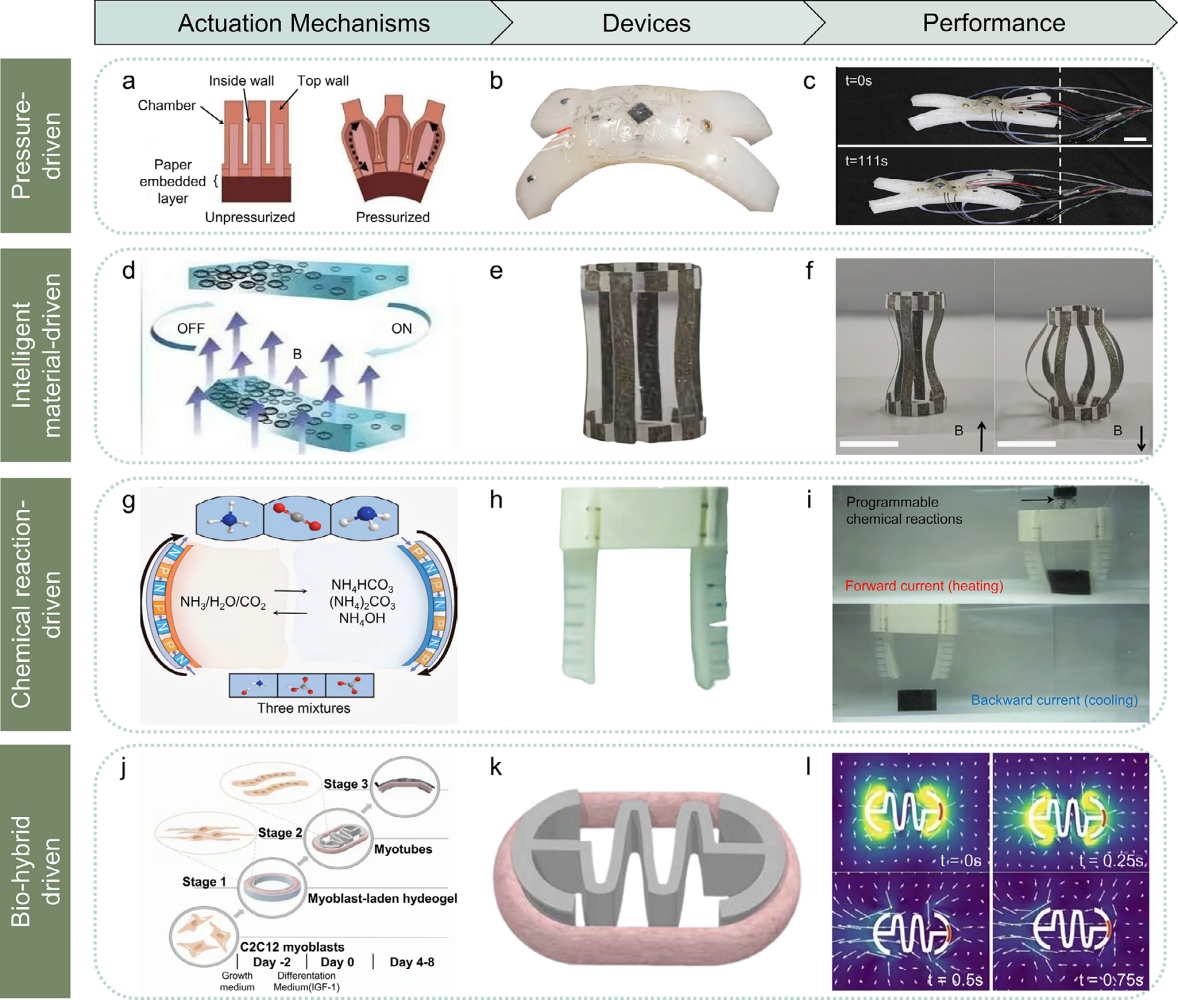
Actuation mechanisms and examples of soft robots. a–c) Pressure‐driven actuators. (a) Schematic of a pneumatic network actuator, where solid arrows indicate actuator regions, and dashed arrows show areas of expansion under pressure.^[^ ^32^ ^]^ Reproduced with permission.^[^ ^32^ ^]^ Copyright 2014, John Wiley & Sons. (b) Physical image of a pneumatic soft robot.^[^ ^33^ ^]^ (c) Optical image of the walking gait of a pneumatic soft robot.^[^ ^33^ ^]^ Reproduced with permission.^[^ ^33^ ^]^ Copyright 2024, AAAS. d–f) Intelligent material‐responsive actuators. (d) Magnetic‐responsive soft actuator made of flexible intelligent magnetic material, driven by non‐uniformly distributed magnetic particles within a polymer matrix.^[^ ^7^ ^]^ Reproduced with permission.^[^ ^7^ ^]^ Copyright 2020, John Wiley & Sons. (e) Physical image of a 3D geometrical soft robot with non‐uniform magnetization.^[^ ^37^ ^]^ (f) Optical images of the 3D soft robot before and after magnetic stimulation, created using hybrid magnetization assembly.^[^ ^37^ ^]^ Reproduced with permission.^[^ ^37^ ^]^ Copyright 2022, AAAS. g–i) Chemically driven actuators. (g) Schematic of the working mechanism of chemically driven actuators, involving reversible reactions in ammonia, carbon, and organic salt systems.^[^ ^56^ ^]^ (h) Soft robotic gripper based on chemically driven actuation.^[^ ^56^ ^]^ (i) Optical image of the soft robotic gripper capturing and moving objects underwater.^[^ ^56^ ^]^ Reproduced with permission.^[^ ^56^ ^]^ Copyright 2024, John Wiley & Sons. (j–l) Bio‐hybrid‐driven actuators. j) Schematic of the fabrication process of a skeletal muscle‐based biohybrid actuator. C2C12 myoblasts were cultured and embedded into a hydrogel scaffold, followed by fusion into aligned myotubes over several days under a differentiation medium containing IGF‐1.^[^ ^59^ ^]^ k) 3D illustration of a ring‐shaped biohybrid swimmer composed of muscle‐laden hydrogel anchored to a serpentine spring skeleton, enabling self‐induced mechanical stimulation during maturation and actuation.^[^ ^59^ ^]^ l) Time‐lapse velocity field images of the biohybrid swimmer actuated at 5 Hz, showing directional swimming driven by muscle contraction without external mechanical input. The robot achieved speeds up to 800 µm s^−1^ (3 body lengths per second), outperforming previous muscle‐based swimmers.^[^ ^59^ ^]^ Reproduced with permission.^[^ ^59^ ^]^ Copyright 2021, AAAS.

Understanding actuator structures and driving strategies of soft robots is critical for advancing bioinspired design, enhancing performance, and expanding functional diversity. Four common driving strategies for soft robots are demonstrated, including pressure‐driven, intelligent material‐driven, chemical reaction‐driven, and bio‐hybrid‐driven mechanisms as shown in **Figure** **3**. We discussed the mechanisms, advantages, limitations, and recent advancements of each strategy, shedding light on the latest developments and challenges in soft robotics. By studying these driving methods, it aims to underscore the field's progress and pave the way for integrating intelligent driving systems in the future, advancing the development of next‐generation intelligent actuation systems.

### 3.1 Pressure‐Driven Actuation

Pressure‐driven actuation stands as a prevalent strategy in the field of soft robotics, offering high flexibility and adaptability. This strategy involves embedding various fluid channels, such as cylindrical, wrinkled, or helical structures, into elastic materials. Additionally, constraint layers are strategically placed to regulate deformation, effectively mirroring the movements of soft‐bodied organisms.^[^ ^28^, ^29^ ^]^ For instance, certain soft organisms execute actions like contraction, expansion, and bending through a blend of muscular and fluid interactions, a phenomenon replicated by the pressure‐driven strategy.

Pressure‐driven systems typically fall into two primary types: pneumatic and hydraulic actuation. Pneumatic actuation relies on compressed gas for power, including deformation through gas flow within elastic channels, making it well‐suited for applications requiring quick response and lightweight design. In contrast, hydraulic actuation uses liquid as the driving medium, delivering increased force and stability, ideal for situations demanding robust load capacity and precise control. While pneumatic systems are simpler and lighter albeit less effective in load handling and precision control, hydraulic systems offer superior force and control precision at the cost of complexity and higher energy consumption.

#### 3.1.1 Pneumatic Actuation

Soft pneumatic actuators (SPAs) are flexible mechanical devices composed of elastic materials such as rubber or silicone, with embedded cavity channels that facilitate structural deformation through controlled air pressure (Figure 3a). By inflating air chambers to mimic biological muscle movement, SPAs combine a lightweight structure with efficient energy conversion, enabling complex motions such as bending, stretching, and rotation in demanding environments.^[^ ^29^, ^32^ ^]^ Despite challenges related to control precision, gas leakage risks, and material durability, these actuators offer significant advantages in precision operations and adaptive tasks that require softness, flexibility, and environmental compatibility.

Pneumatic artificial muscles (PAMs), a classic type of SPA exemplified by the McKibben actuator, operate by injecting air into flexible materials, inducing expansion or contraction through pressure changes to replicate biological muscle movement.^[^ ^3^ ^]^ De Pascali et al. improved this design with the GRACE structure, which achieves expansion‐contraction coupling through geometric optimization, eliminating the need for traditional tensile components while enabling scalable sizing and adjustable driving forces across a broad range.^[^ ^30^ ^]^ Further innovations include Zhang et al.’s SPA‐based gripper integrated with triboelectric sensors, which utilizes pulse signal modulation for the precise handling of fragile objects.^[^ ^31^ ^]^ Stephanie et al. expanded SPA functionality by embedding a highly stretchable Arduino Pro Mini (>300% strain) into a soft robot (Figure 3b), enabling autonomous gait generation through I2C‐controlled pressure regulation and computational decision‐making (Figure 3c).^[^ ^33^ ^]^ These advancements underscore the increasing adaptability of SPAs in multifunctional systems involving force modulation, sensing integration, and embedded intelligence.

#### 3.1.2 Hydraulic Actuation

Hydraulic soft robots use pressurized liquids within flexible materials to achieve motion, providing high force output, precise control, and stability through fluid‐elastic actuators composed of stretchable fluid channels and non‐stretchable constraint layers.^[^ ^3^ ^]^ While their ability to perform directional bending under pressure makes them well‐suited for high‐precision applications such as medical robotics, challenges include system complexity due to liquid management, leakage risks, and high energy consumption. Recent advancements have focused on self‐repair, motion modeling, and multimodal actuation: Tang et al. developed a self‐protecting actuator with an embedded soft electrohydraulic pump and self‐healing electrorheological material, enabling wireless operation and damage repair,^[^ ^34^ ^]^ while Xie et al. engineered a fiber‐reinforced three‐chamber hydraulic actuator capable of multi‐degree‐of‐freedom motion through axial elongation constraints.^[^ ^35^ ^]^ Kurumaya et al. integrated hydraulic corrugated grippers with coiled rotational actuators on underwater robots, allowing for delicate coral sampling without damage.^[^ ^36^ ^]^ These innovations highlight the growing versatility of hydraulic actuation in balancing force intensity with environmental sensitivity, though challenges in dynamic modeling and fluid control remain.

### 3.2 Intelligent Material‐Driven Actuation

Intelligent material‐driven actuation, also known as stimuli‐responsive actuation, enables reversible motion or shape transformation in actuators through physical or chemical changes triggered by external stimuli, such as temperature, pH, light, electricity, magnetism, or humidity.^[^ ^7^ ^]^ The mechanism relies on the intrinsic sensitivity of materials to stimuli, inducing structural or property changes that drive mechanical motion. Figure 3d illustrates a polymer‐based actuator with unevenly distributed magnetic particles embedded in its matrix. Under a magnetic field, localized deformation occurs due to spatially varying magnetic forces. Building on this concept, Dong et al. (Figure 3e) designed an actuator with programmed magnetization patterns using NdFeB particles. By controlling magnetic domain alignment, the actuator achieves directional motion, such as radial contraction or expansion (Figure 3f).^[^ ^37^ ^]^ These magnetically responsive systems have been applied in targeted biomedical devices, including capsules and drug delivery platforms.

Inspired by biological systems such as humidity‐responsive pinecones and light‐triggered plant tendrils, stimuli‐responsive actuators combine flexibility, zero external power requirements, and adaptability to complex environments. However, challenges remain regarding response speed, control precision, and environmental dependency.^[^ ^7^ ^]^

**Figure 4:**
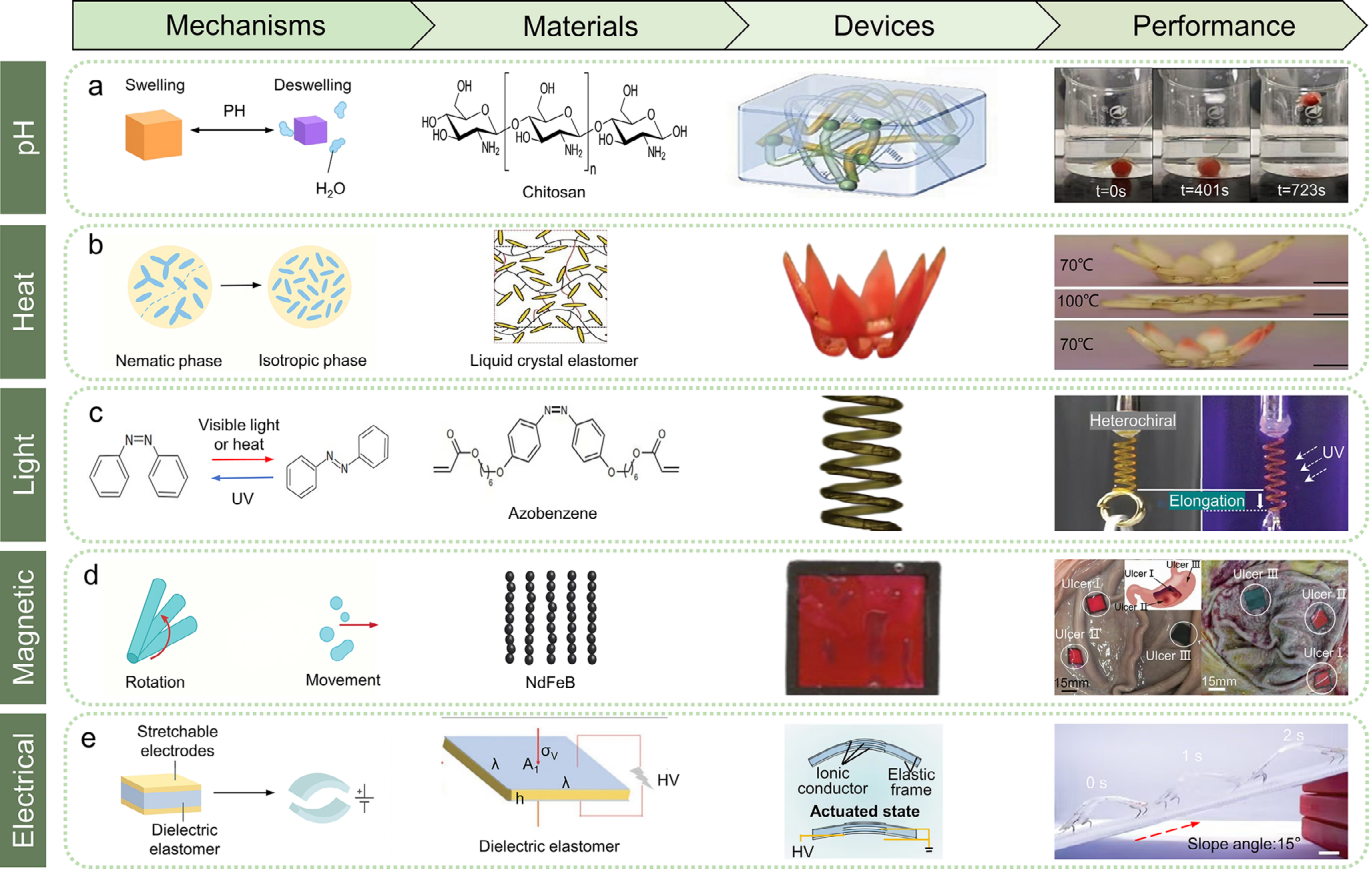
Illustration of the mechanisms, materials, devices, and actuation performance of intelligent material‐driven soft robots. a) pH‐responsive actuation: chemical structure of chitosan and schematic of polyvinyl alcohol/sodium alginate/chitosan hydrogel‐based actuator. The right panel shows a pH‐sensitive gripper wrapping and lifting a clay ball, demonstrating volume change‐induced gripping behavior under varying pH (t = 0 s, 401 s, 723 s).^[^ ^38^ ^]^ Reproduced with permission.^[^ ^38^ ^]^ Copyright 2024, Elsevier. b) Thermally responsive actuation: actuation is driven by reversible phase transition between nematic and isotropic phases under temperature stimuli. Includes the structure of liquid crystal elastomers,^[^ ^63^ ^]^ and a flower‐shaped actuator embedded with thermochromic dyes, which shows blooming behavior with temperature change from 70 °C to 100 °C, and reversibility upon cooling.^[^ ^41^ ^]^ Reproduced with permission.^[^ ^63^ ^]^ Copyright 2020, Elsevier. Reproduced with permission.^[^ ^41^ ^]^ Copyright 2022, Royal Society of Chemistry. c) Light‐responsive actuation: based on azobenzene‐driven photoisomerization and photothermal effects. The left panel shows the reversible chemical structure transition of azobenzene; the middle panel shows an azobenzene‐functionalized semicrystalline liquid crystal elastomer spring actuator; the right panel demonstrates UV‐induced elongation of heterochiral springs with visible displacement and real‐time deformation.^[^ ^43^ ^]^ Reproduced with permission.^[^ ^43^ ^]^ Copyright 2024, John Wiley & Sons. d) Magnetically responsive actuation: actuation enabled by applied magnetic fields causing rotation or displacement. Illustrated are the NdFeB microstructure,^[^ ^85^ ^]^ a magnetic multilayer robot, and its targeted adhesion to gastric mucosa of pig stomachs, demonstrating effective locomotion and anchoring under dynamic conditions (Ulcer I–III).^[^ ^44^ ^]^ Reproduced with permission.^[^ ^85^ ^]^ Copyright 2022, American Chemical Society. Reproduced with permission.^[^ ^44^ ^]^ Copyright 2024, Springer Nature. e) Electrically responsive actuation: deformation driven by electric fields through dielectric elastomers.^[^ ^171^ ^]^ The figure includes a schematic of stretchable dielectric elastomer structure and actuation mechanism. The right panel shows an amphibious soft robot climbing a slope, with visible bending deformation over time (slope angle = 15°, t = 0–2 s), indicating fast and reversible bending under high voltage stimulation.^[^ ^47^ ^]^ Reproduced with permission,^[^ ^171^ ^]^ Copyright 2020, Elsevier. Reproduced with permission.^[^ ^47^ ^]^ Copyright 2023, John Wiley & Sons.

Recent advances in soft robotics driven by intelligent materials are categorized based on stimulus type: pH,^[^ ^38^, ^39^ ^]^ thermal,^[^ ^40^, ^41^ ^]^ light,^[^ ^42^, ^43^ ^]^ magnetic,^[^ ^44^, ^45^, ^46^ ^]^ and electrical response.^[^ ^47^, ^48^ ^]^ **Figure** **4** summarizes their working mechanisms, material designs, applications, and performance metrics.

#### 3.2.1 pH‐Responsive Actuators

In nature, organisms adjust their morphology or movement in response to environmental pH changes. For example, plant roots and leaves modify growth patterns under acidic or alkaline conditions, aquatic plants such as duckweed and algae adapt to pH fluctuations through morphological changes, and marine organisms like shellfish and corals experience slowed shell growth in acidic environments due to inhibited calcium carbonate deposition. Inspired by these natural responses, pH‐responsive soft actuators have been developed, utilizing the pH‐dependent ionization of functional groups and electrostatic repulsion among charges.

A representative example is the double‐network polyvinyl alcohol/sodium alginate/chitosan (PSCS) hydrogel developed by Xu et al.,^[^ ^38^ ^]^ created by diffusing chitosan into a polyvinyl alcohol (PVA) and sodium alginate semi‐interpenetrating network. The incorporation of chitosan increases structural density, reduces porosity, and enhances crystallinity and mechanical strength. In experiments, a cross‐shaped PSCS clamp successfully grasped a 0.3 g clay ball in a pH 2 solution within 723 s and lifted it under external force, maintaining functionality even after drying (Figure 4a). This demonstrates its strong pH responsiveness, reusability, and environmentally friendly preparation without toxic chemicals, contributing to the advancement of flexible actuator design.

These actuators efficiently convert ambient chemical energy into mechanical work without requiring external power, enabling autonomous operation in wireless settings. For example, by leveraging natural pH gradients in human organs, they can be programmed for precise drug delivery, automatically triggering actions under specific pH conditions to ensure targeted spatiotemporal release.

#### 3.2.2 Thermo‐Responsive Actuators

While pH‐driven actuators exhibit impressive performance, their dependence on aqueous or humid environments limits their applicability in dry conditions, prompting interest in thermo‐responsive alternatives. These heat‐actuated systems, which respond to exothermic reactions, photothermal effects, Joule heating, or magnetocaloric stimuli, enable versatile actuation mechanisms and significant shape and volume changes.^[^ ^7^ ^]^

Biological systems, which adapt through reversible movements, serve as inspiration for actuator design. In the context of thermal responsiveness, shape‐memory polymers (SMPs) are widely explored for their bidirectional reversible shape‐memory effects. Temperature shifts induce conformational changes in molecular chains, allowing SMPs to transition between fixed and recovered states via thermally sensitive functional groups or crosslinks.^[^ ^1^, ^40^ ^]^

Liquid crystal elastomers (LCEs) achieve reversible thermal actuation through nematic‐isotropic phase transitions, driven by molecular rearrangement and elasticity. Li et al. designed a flower‐like thermochromic LCE structure (Figure 4b), demonstrating dual shape‐color reversibility. At 52 °C, its color shifts from red to colorless; at 62 °C, the LCE undergoes a phase transition, flattening the 3D structure into a 2D form. Cooling below the LCE's clearing temperature and the dye solvent's melting point restores both its original color and 3D shape, mimicking natural blooming cycles.^[^ ^41^ ^]^

The phase transition characteristics of LCEs at different temperatures enable their broad application in bioinspired actuators and robotics, including iris‐like apertures, artificial muscles, and caterpillar‐like soft robots. Although LCE‐based thermo‐responsive actuators offer advantages such as fast response and programmability, they also have limitations, including high demands for temperature precision, slower response speeds, increased energy consumption, poor environmental stability, and complex manufacturing processes.

#### 3.2.3 Photo‐Responsive Actuators

Light is a highly promising stimulus for bio‐inspired soft actuators due to its adjustability, non‐invasiveness, and precise spatiotemporal control.^[^ ^42^ ^]^ Actuators respond to wavelengths ranging from ultraviolet (UV) to near‐infrared through photothermal or photochemical mechanisms, mimicking natural motion strategies such as cell‐triggered movement without mechanical linkages. For example, azobenzene‐based LCEs enable photochemical actuation through trans‐cis isomerization under UV light (300–400 nm) and reversible cis‐trans recovery under visible light (425–500 nm). Seo et al. demonstrated a semi‐crystalline azobenzene‐based LCE spring with 60 percent elongation and a work output of 15 kJ·m⁻^3^ under UV light, achieving three times the elongation and twice the work output of previous azobenzene actuators (Figure 4c).^[^ ^43^ ^]^ This system operates isothermally in various environments, including underwater, without continuous energy input due to its photochemical locking mechanism. Structural chirality dictates the actuation direction: homochiral springs contract under UV light and expand under visible light, while heterochiral springs exhibit the opposite behavior. Spiropyran derivatives provide visible‐light responsiveness through ring‐opening isomerization, reversibly switching between open‐ and closed‐ring states. This process alters molecular conformation and hydrogel network properties, enabling light‐triggered swelling and contraction. Consequently, UV‐ and visible‐light‐controlled soft robots such as grippers and crawlers can achieve large‐stroke axial and radial deformation for specialized environments. Although these light‐driven actuators have broad application potential, they still face challenges such as slower response times and limited diversity in actuation modes.

#### 3.2.4 Magnetic‐Responsive Actuators

Magnetic‐responsive soft robotic actuators leverage remotely controlled magnetic fields to manipulate objects without physical connections. These systems use soft composites embedded with magnetic particles such as NdFeB in polydimethylsiloxane (PDMS), enabling programmable magnetization patterns for controlled deformation and motion. Each particle acts as a magnetic dipole, with external fields exerting localized forces and torques to achieve complex actuation. This untethered operation is particularly advantageous for medical applications, including targeted drug delivery and intravascular procedures, where confined spaces require precise, field‐tunable positioning.

Chen et al. demonstrated this potential with a multilayer magnetic soft robot (Figure 4d) composed of a PDMS and ferromagnetic particle substrate, along with carbopol‐based adhesive layers containing poloxamer and hydroxypropylmethylcellulose additives. The tri‐layer structure enabled controlled translational and rolling motions, as well as on‐demand separation for tissue attachment. Experiments validated its targeted adhesion capabilities on ex vivo gastric tissues, in liquid‐filled stomach models, and through in vivo multi‐target trials, highlighting its clinical potential for gastrointestinal interventions.^[^ ^44^ ^]^

The adaptive deformation of these composites mimics biological systems such as Escherichia coli and jellyfish, suggesting broad applications in soft robotics and biomedical devices.^[^ ^45^, ^46^ ^]^ However, current magnetic field generation systems suffer from bulkiness, inherently limiting portability. Under strong or high‐frequency magnetic fields, control precision and energy efficiency are both reduced. In complex biological environments, response speed and magnetic penetration depth remain constrained. Additionally, a critical trade‐off persists between achieving strong magnetic performance and maintaining optimal biocompatibility. Overcoming these multifaceted challenges is pivotal to translating this technology into practical real‐world applications.

#### 3.2.5 Electro‐Responsive Actuators

Electric actuators, while essential in traditional robotics for their precision and durability, face compatibility challenges in soft robots due to their rigid components. Recent advancements have introduced electrically responsive actuators optimized for soft robotics, leveraging direct electrical‐to‐mechanical energy conversion and seamless integration with electronic systems.^[^ ^48^ ^]^ Among these, DEAs are particularly notable, utilizing Coulomb interactions between flexible electrodes and elastomer layers to achieve rapid, low‐power deformation with a large strain capacity.

Cheng et al. demonstrated the potential of DEAs with a five‐layer amphibious soft robot (Figure 4e) that incorporated environmentally stable ion‐electrode materials. Without additional encapsulation, it maintained a locomotion speed of 6.7 cm s^−1^ on a 15° slope and operated effectively in both land and water environments.^[^ ^47^ ^]^ However, DEAs remain susceptible to dynamic failure modes such as dielectric breakdown and pull‐in effects.

Other types of electrically responsive actuators include electroactive polymers, which enable large deformations but suffer from slower response times and stability issues, and electrochemical materials such as ion gels, which perform well in wet conditions despite their sensitivity to environmental fluctuations.^[^ ^7^ ^]^ Material selection depends on application‐specific requirements, balancing flexibility, response speed, and environmental resilience.

#### 3.2.6 Multi‐Stimulus‐Responsive Actuators

Despite advancements in single‐stimulus‐driven actuators, multi‐stimulus‐responsive systems have emerged to integrate diverse stimuli such as electrical, thermal, light, and humidity to enhance multifunctionality and flexibility. These actuators, often utilizing stimuli‐responsive materials like hydrogels,^[^ ^49^, ^50^ ^]^ enable precise control in complex environments due to their adaptability and compatibility with various applications.

However, several challenges remain, including material response interference, system complexity, high fabrication, and control costs, and reduced response speed and stability under simultaneous multi‐stimuli exposure. In particular, stimulus interference arises from overlapping activation pathways, such as simultaneous thermal and electrical inputs altering ionic mobility or crosslinking behavior, which may lead to undesired deformation or delayed actuation.^[^ ^51^ ^]^ For example, in hydrogel‐based systems, heat‐induced shrinking may counteract electrically driven expansion, reducing actuator predictability.^[^ ^52^ ^]^

To address these challenges, current research is exploring strategies such as spatially separating response domains within composite materials,^[^ ^53^ ^]^ designing orthogonal stimulus‐response networks,^[^ ^54^ ^]^ and employing hierarchical control algorithms to sequentially trigger specific responses.^[^ ^55^ ^]^ These approaches aim to minimize cross‐talk, improve reliability, and enable programmable, task‐specific actuation under complex environmental conditions.

### 3.3 Chemical Reaction Actuation

Chemically driven actuators generate mechanical deformation through chemical reactions such as redox and acid‐base processes, enabling operation without external power sources (Figure 3g). This makes them particularly suitable for autonomous systems or chemically and biologically active environments. Notable examples include Wehner et al.’s fully soft Octobot,^[^ ^12^ ^]^where hydrogen peroxide decomposition via a platinum catalyst generates gas‐driven movement through 3D‐printed microchannels, and Qu et al.’s programmable reactions utilizing thermoelectric materials (Figure 3h) and reversible reactants such as ammonia and carbon‐based systems to achieve precise gas pressure control of ≈6 MPa for underwater soft grippers (Figure 3i).^[^ ^56^ ^]^ Other approaches harness gas combustion, such as nitrous oxide‐propane^[^ ^57^ ^]^ or butane‐oxygen mixtures,^[^ ^62^ ^]^ to power soft robots.

However, these actuators face several limitations, including slow response speeds, limited reaction controllability, material degradation from repeated reactions, dependence on specific chemicals, and environmental sensitivity to temperature and humidity. Despite these challenges, advances in chemical programmability and material design continue to drive progress in soft robotics, offering potential breakthroughs in efficiency and adaptability.

### 3.4 Bio‐Hybrid Actuation

Bio‐hybrid actuation mechanisms represent an emerging class of soft robotic strategies in which living cells, such as cardiomyocytes or skeletal muscle tissues, are integrated into synthetic frameworks to generate autonomous, lifelike motion.^[^ ^61^ ^]^ By leveraging the intrinsic contractility of muscle cells and their interaction with compliant mechanical scaffolds, these systems enable soft robots to perform complex behaviors such as bending, swimming, and pulsing. Unlike conventional actuators that rely on external power sources such as batteries or pneumatic pumps, bio‐hybrid systems are self‐actuating, capable of operating in a fully autonomous manner. In addition, their responsiveness to environmental stimuli—such as temperature, light, or chemical gradients—offers fine‐grained control, while their inherent biocompatibility makes them particularly suitable for in vivo applications, including drug delivery and minimally invasive surgical tools. A representative example is the skeletal muscle–driven swimmer developed by Guix et al., which integrates a 3D‐printed serpentine spring skeleton with muscle tissue to enable self‐induced mechanical stimulation and enhanced force output during maturation (Figure 3j).^[^ ^59^ ^]^

A seminal example of this approach is the medusoid robot developed by Nawroth et al., which employed cardiomyocytes seeded onto an elastic scaffold to mimic the propulsion of a jellyfish.^[^ ^58^ ^]^ Further experiments by Guix et al. showed that the biohybrid swimmer could perform directional swimming and coasting depending on its position in the medium (Figure 3k). At 5 Hz electrical stimulation, it reached speeds of 800 µm s^−1^ (three body lengths per second). Programmable asymmetry in the skeleton further enabled repeatable turning and trajectory control (Figure 3l), demonstrating efficient actuation and adaptive locomotion in confined aquatic environments.^[^ ^59^ ^]^ In parallel, Yalikun et al. developed a microscale swimming robot driven by dorsal vessel tissue extracted from inchworm larvae. Without any external stimulus, the robot achieved autonomous motion at a speed of 11.7 µm s^−1^. Notably, the system maintained cyclic pumping and movement over several days under varying temperature and humidity, demonstrating high biomechanical stability and potential for use in untethered in vivo applications.^[^ ^60^ ^]^ These representative studies demonstrate the feasibility and promise of integrating living tissues with engineered frameworks to achieve autonomous, efficient, and environmentally responsive motion, paving the way for next‐generation biohybrid soft robots in biomedical and microscale applications.

Despite these advancements, bio‐hybrid actuators still face challenges including limited lifespan, difficulty in integration with control systems, and sensitivity to environmental changes. Looking forward, developments in stem‐cell derived tissues, neuromuscular interfacing, and biocompatible scaffolding are expected to enhance stability, scalability, and functionality, advancing their applicability in implantable systems, autonomous microbots, and biologically integrated machines.

## 4 Materials Selections for Soft Robotics

**Figure 5:**
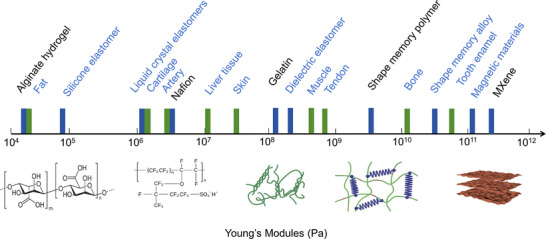
Comparison of the approximate Young's modulus between materials used in soft robotics and biological materials. The chemical formulas or structural diagrams of alginate hydrogel, Nafion, gelatin,^[^ ^66^ ^]^ shape memory polymers,^[^ ^68^ ^]^ and MXene materials^[^ ^86^ ^]^ are shown below the axis. Reproduced with permission.^[^ ^66^ ^]^ Copyright 2020, Springer Nature. Reproduced with permission.^[^ ^68^ ^]^ Copyright 2021, John Wiley & Sons. Reproduced with permission.^[^ ^86^ ^]^ Copyright 2025, Elsevier.

Soft materials serve as the foundation of soft robotics, enabling flexible and adaptive systems through bioinspired compliance.^[^ ^63^, ^64^ ^]^ Material selection plays a critical role in determining mechanical performance and environmental adaptability, with Young's modulus, a key stiffness parameter, governing deformation behavior.^[^ ^6^ ^]^ As shown in **Figure** **5**, Young's modulus of these materials closely aligns with that of natural biological tissues, enhancing bioinspired functionality and interaction capabilities. Materials with an appropriate Young's modulus are increasingly gaining attention in the field of soft robotics and are becoming a key foundational material driving the advancement of soft robot technologies. The development of programmable materials enabling adaptive morphology and tunable mechanical properties marks a key step toward next‐generation soft robotics. By allowing dynamic responses to complex environments, these materials support reconfigurable designs and smarter actuation, paving the way for more intelligent robotic systems.

### 4.1 Materials with Low Young's Modulus

Materials with low Young's modulus are particularly important for soft robotics due to their inherent flexibility, deformability, and compatibility with biological systems. Such materials enable large, reversible deformations under low mechanical stress, which is essential for safe interaction with humans and adaptive behavior in unstructured environments. For instance, materials with low Young's modulus, such as hydrogels,^[^ ^66^, ^70^, ^71^, ^72^, ^73^ ^]^ silicones,^[^ ^78^ ^]^ and liquid metals,^[^ ^79^, ^80^ ^]^ offer excellent flexibility and deformability, enabling soft robots to achieve complex shape changes and movements in response to external stimuli. Hydrogels, in particular, represent a diverse group of low‐modulus, water‐swollen polymer networks with excellent biocompatibility and responsiveness. They can be tuned to respond to stimuli such as temperature, pH, and ionic concentration, making them highly adaptable for soft robotic actuation and sensing.

#### 4.1.1 Stimuli‐Responsive Hydrogels

Hydrogels are a prominent class of low‐modulus materials composed of water‐rich, crosslinked polymer networks that exhibit high flexibility, biocompatibility, and environmental responsiveness. Among them, alginate^[^ ^73^ ^]^ and gelatin,^[^ ^66^ ^]^ both derived from natural sources, are widely utilized in soft robotics. Alginate hydrogels form ionically crosslinked networks that respond to hydration and ionic concentration, enabling tunable shape transformation under mild conditions.^[^ ^73^ ^]^ Gelatin, with its reversible sol‐gel transition and responsiveness to temperature, humidity, and electric fields, supports bioinspired deformation and thermally driven actuation.^[^ ^66^ ^]^ Their excellent biocompatibility and biodegradability make them ideal candidates for biomedical and human‐interactive applications. However, both materials face limitations in mechanical strength and long‐term stability, which can be mitigated through composite design or structural reinforcement.

In addition to natural hydrogels, synthetic stimuli‐responsive hydrogels such as poly(N‐isopropylacrylamide) (PNIPAM)^[^ ^74^, ^75^ ^]^ and poly(acrylic acid) (PAAc)^[^ ^76^, ^77^ ^]^ are extensively studied for their sharp and reversible responsiveness. PNIPAM exhibits temperature‐dependent phase transitions near 32 °C,^[^ ^75^ ^]^ allowing it to shrink or swell with minimal thermal input, while PAAc undergoes pH‐sensitive volume changes, enabling fine control of deformation in varying environments.^[^ ^77^ ^]^ These classic hydrogels provide valuable platforms for developing programmable, environment‐adaptive actuators in soft robotic systems. Despite their unique responsiveness and tunable softness, hydrogel‐based materials are often limited by low mechanical durability and dehydration under open conditions, highlighting the need for ongoing material optimization.

#### 4.1.2 Silicone Elastomer

Silicone elastomer, a synthetic polymer composed of silicon, oxygen, carbon, and hydrogen, exhibits high thermal stability, elasticity, chemical resistance, biocompatibility, and electrical insulation. Its flexibility and deformability enable bioinspired motion in soft robotics, often functioning as a protective yet tactile‐friendly outer layer. When integrated with pneumatic or hydraulic systems, it allows precise movement control. The material's durability under extreme temperatures, chemical stability, and sealing capabilities enhance robotic reliability in harsh environments. However, challenges such as limited mechanical strength, restricted tolerance to high temperatures, and gradual degradation under specific conditions require further refinement for advanced applications.^[^ ^78^ ^]^ Despite these limitations, its unique properties highlight its significance in developing resilient and adaptable robotic systems.

### 4.2 Materials with Moderate Young's Modulus

In addition to the materials mentioned in the section on intelligent material responses to actuation, there are many other materials with moderate Young's modulus that can be used to fabricate soft robots. These materials are capable of providing sufficient rigidity for support while maintaining excellent flexibility and deformability, thus enabling effective morphological changes and motion under various external stimuli. Common examples of such materials include Nafion,^[^ ^65^ ^]^ SMPs,^[^ ^67^, ^68^ ^]^ and DE.^[^ ^69^ ^]^ These materials closely match the mechanical compliance of biological tissues and exhibit remarkable adaptability and toughness in scenarios involving high loads and large deformations.

#### 4.2.1 Nafion

Nafion is a high‐performance ion‐exchange membrane material primarily composed of a polytetrafluoroethylene (PTFE) backbone and sulfonic acid group side chains. It boasts exceptional ionic conductivity, chemical stability, and mechanical strength. Thanks to its excellent ion‐conductive and flexible properties, Nafion is widely used in liquid‐driven systems and sensors. Under the influence of electric fields or electrochemical stimuli, Nafion can change shape, thereby driving deformation in robotic structures.^[^ ^65^ ^]^ Additionally, it can be utilized in sensors that are sensitive to humidity or temperature, providing real‐time environmental feedback. However, it can be expensive and may degrade under extreme conditions, such as high temperatures or strong oxidizing environments.

#### 4.2.2 Shape Memory Polymers

SMPs are programmable polymers that recover predetermined shapes in response to stimuli such as temperature, humidity, and light, enabling precise actuation and simplifying robot design through inherent mechanical memory.^[^ ^67^, ^68^ ^]^ Their shape‐memory functionality enhances robotic flexibility while reducing dependence on complex components. However, current limitations, including low strength, slow response, and durability issues, could be mitigated through nanomaterial reinforcement and molecular optimization to meet the demands of advanced applications.

### 4.3 Materials with High Young's Modulus

Materials with a high Young's modulus, such as shape memory alloys (SMAs),^[^ ^81^, ^82^, ^83^, ^84^ ^]^ can provide structural rigidity and are suitable for components that require high mechanical stability. In contrast, magnetic materials^[^ ^85^ ^]^ and MXenes,^[^ ^86^, ^87^ ^]^ while intrinsically stiff, are typically incorporated into soft robotic systems as functional additives or dopants to impart magnetic responsiveness, conductivity, or sensing capability, rather than serving as primary load‐bearing structural elements.

#### 4.3.1 Shape Memory Alloys

SMAs, typically nickel‐titanium‐based compounds, exhibit temperature‐triggered shape recovery, known as the shape memory effect, along with superelastic deformation capabilities.^[^ ^81^, ^82^ ^]^ These properties enable flexible actuation, allowing robots to replicate biological motions such as bending and grasping while adapting precisely to environmental changes.^[^ ^83^, ^84^ ^]^ Despite these advantages, challenges remain, including high costs, susceptibility to mechanical fatigue, processing complexity, and long‐term performance degradation. Current research focuses on improving durability and efficiency through alloy optimization, composite material development, and multiscale manufacturing techniques.

#### 4.3.2 Magnetic Materials

Magnetic materials, which respond to external magnetic fields, enable precise shape deformation and motion control in soft robotics through contactless actuation. By guiding flexible actuators, they reduce mechanical friction and wear, thereby enhancing system reliability.^[^ ^85^ ^]^ When integrated into sensors, they detect magnetic field variations to provide real‐time positional and status feedback, facilitating closed‐loop control. Smart variants further improve adaptability and autonomy, allowing robots to operate with high flexibility and accuracy in complex environments. These properties make magnetic materials essential for advancing intelligent, low‐maintenance soft robotic systems.

Optimizing programmable materials for soft robotics requires strategic selection and customization to meet specific functional demands. For instance, actuation components benefit from materials with a low Young's modulus to ensure high flexibility and dynamic responsiveness, whereas structural elements require greater stiffness for stability. Tunable properties such as stiffness, elasticity, and responsiveness expand design possibilities, enhancing adaptive morphology and functional efficiency.

Future advancements in intelligent soft robotics will focus on materials with multifunctional flexibility, self‐adaptability, and embedded sensing to enable precise control in unstructured environments. Innovations in programmable materials and additive manufacturing will drive the development of autonomous systems with embodied cognition, facilitating transformative applications in biomedicine, environmental exploration, and human‐machine interfaces.

## 5 Manufacturing Methods of Soft Robotics

**Figure 6:**
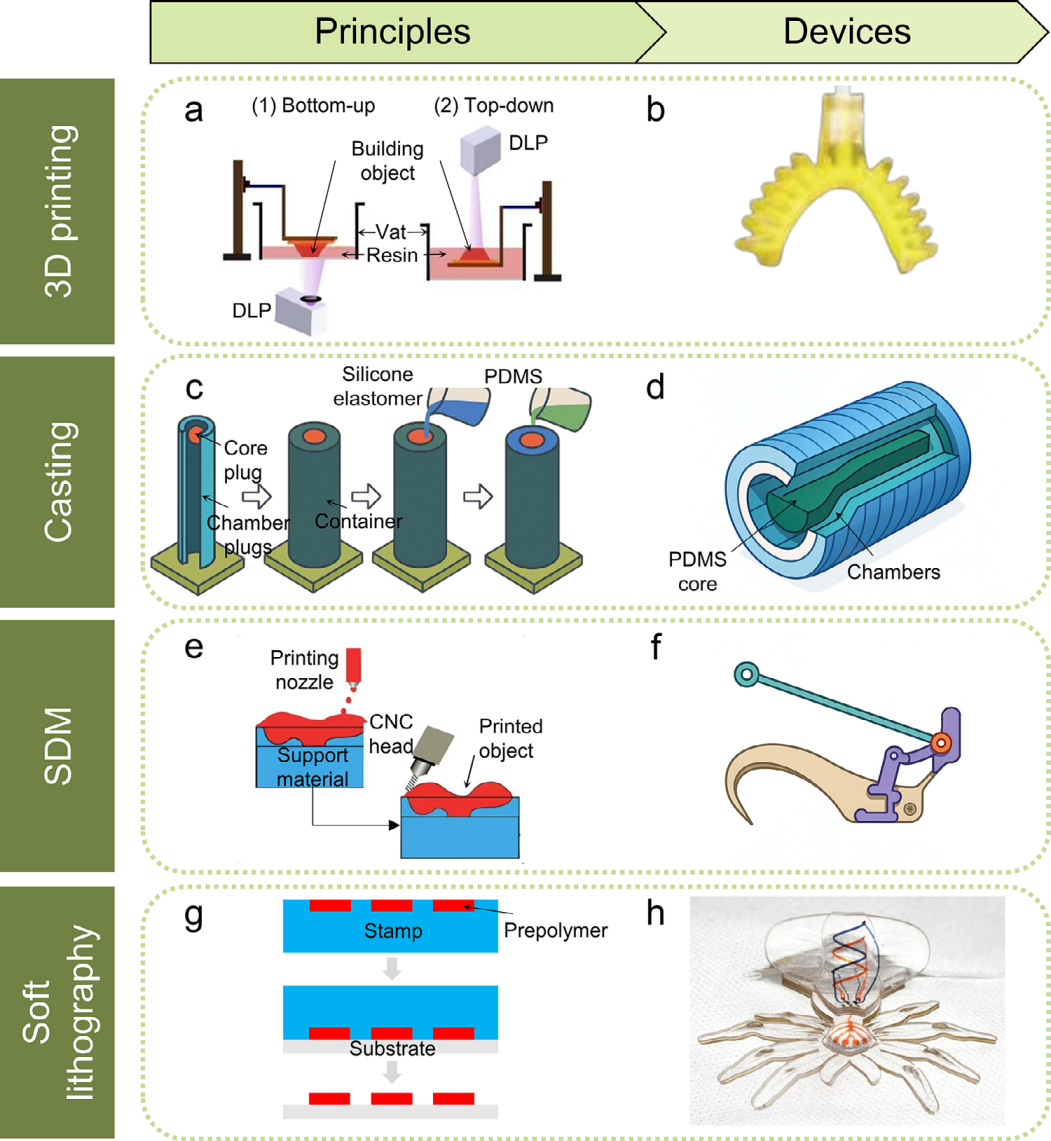
Fabrication principles and devices of soft robots. a) Schematic of the DLP technology principle, including both bottom‐up and top‐down configurations.^[^ ^92^ ^]^ Reproduced with permission.^[^ ^92^ ^]^ Copyright 2022, Springer Nature. b) A pneumatic soft gripper fabricated using DLP technology with deep eutectic solvent‐based resin.^[^ ^94^ ^]^ Reproduced with permission.^[^ ^94^ ^]^ Copyright 2024, John Wiley & Sons. c) Schematic of the casting method principle. d) A 3D soft robotic arm molded using silicone and polylactic acid molds. e) Schematic of the SDM process.^[^ ^93^ ^]^ Reproduced with permission.^[^ ^93^ ^]^ Copyright 2024, Taylor & Francis. f) A microneedle gripper fabricated through the SDM process. g) Principle schematic of the µTM technique in soft lithography.^[^ ^88^ ^]^ Reproduced with permission.^[^ ^88^ ^]^ Copyright 2024, Springer Nature. h) A spider‐shaped robot developed using multilayer soft lithography techniques with multilayer silicone elastomers.^[^ ^102^ ^]^ Reproduced with permission.^[^ ^102^ ^]^ Copyright 2018, John Wiley & Sons.

Soft robot fabrication relies heavily on material selection and actuation mechanisms, as their mechanical properties—such as elasticity, rigidity, and strength—directly influence motion efficiency, durability, and environmental adaptability.^[^ ^88^, ^89^ ^]^ Manufacturing techniques must align with these material properties and actuation requirements in a multiscale fashion. For example, compliant actuators require high elasticity, while structural elements prioritize rigidity. As shown in **Figure** **6**, Common manufacturing methods include 3D printing,^[^ ^91^, ^92^, ^93^, ^94^, ^95^ ^]^ casting,^[^ ^96^ ^]^ shape deposition manufacturing (SDM),^[^ ^97^, ^98^, ^99^, ^100^ ^]^ and soft lithography.^[^ ^101^, ^102^, ^103^ ^]^ Each approach must balance material compatibility, processing precision, and functional performance to meet diverse application needs. This section highlights these key methods, emphasizing their roles in developing high‐performance, adaptable robotic systems.

### 5.1 3D Printing Technology

3D printing, also known as additive manufacturing, constructs objects by sequentially layering materials, distinguishing them from traditional subtractive methods such as cutting or milling. This technology enables precise geometric control using digital models, minimizes material waste, and facilitates the fabrication of complex structures. Common techniques include fused deposition modeling (FDM), stereolithography (SLA), selective laser sintering (SLS), direct ink writing (DIW), and digital light processing (DLP).^[^ ^91^, ^95^ ^]^

FDM operates by extruding heated thermoplastic filaments through a nozzle, depositing material layer by layer onto a build platform. The nozzle follows a programmed path to shape the 3D structure, completing the process once the object is fully formed. In contrast, SLA uses UV lasers to cure photosensitive resin one layer at a time. After each layer solidifies according to the sliced model data, the build platform lowers, repeating the process until the final structure is achieved.^[^ ^93^ ^]^

SLS employs a laser to sinter powdered materials, fusing particles just above their melting point. After sintering each layer, the platform lowers, a fresh layer of powder is spread, and the process continues. Once printing is complete, the unsintered powder is removed, revealing the finished part.^[^ ^92^ ^]^ DIW extrudes high‐viscosity inks—such as polymers, ceramics, or biomaterials—through a nozzle, depositing layers with precise control over extrusion volume, speed, and trajectory. Additional drying or curing steps finalize the object.

DLP utilizes a digital micromirror device (DMD) to project light patterns onto photosensitive resin, curing it layer by layer. It operates in two primary configurations: bottom‐up, where the build platform rises from the resin vat, and top‐down, where the light source is positioned above the resin while the platform is submerged (Figure 6a). Huang et al. demonstrated the potential of DLP by printing a pneumatically driven soft gripper using deep eutectic solvent (DES) resin. The photopolymerized DES resin formed a tough, self‐healing ion gel (Figure 6b), allowing the gripper to recover functionality even after severe damage, highlighting its durability and potential for actuation and large‐scale repair.^[^ ^94^ ^]^

Multi‐material 3D printing integrates diverse materials either simultaneously or alternately, enhancing functionality in applications such as healthcare, soft robotics, and electronics. FDM achieves this through multi‐nozzle systems, filament switching, or soluble support materials like PVA. Inkjet printing employs multiple printheads to deposit different materials layer by layer, followed by curing. DIW combines structural, conductive, and support materials through multi‐nozzle designs or ink mixing. Embedded 3D printing directly integrates functional components such as sensors and electronics during fabrication. For instance, Wehner et al. embedded pneumatic actuator networks, fuel storage units, and catalytic chambers into a soft robotic body using multi‐material deposition. This approach enabled programmable assembly, advancing fully autonomous soft robots.^[^ ^12^ ^]^

3D printing offers unparalleled design freedom, rapid customization, and reduced material waste, contributing to sustainability. Its ability to integrate sensors, actuators, and circuits into monolithic structures is driving innovation in soft robotics and smart devices. Advances in materials, printing speed, and precision are expanding applications in personalized medicine, aerospace, and sustainable manufacturing. This technology is poised to revolutionize industries through functional integration and tailored solutions.^[^ ^91^, ^93^ ^]^

### 5.2 Casting

Casting, a fundamental manufacturing technique, involves injecting flexible materials such as silicones and PDMS into molds and solidifying them through thermal processes (Figure 6c) to create lightweight, adaptable robotic components. Its precision and cost‐effectiveness make it well‐suited for producing simple yet functional parts, including flexible joints, grippers, and actuators. However, challenges include long curing times, mold complexity, and material constraints. Casting is typically classified into single‐stage methods for basic designs and multi‐stage methods for complex geometries, multi‐material integration, or pneumatic actuators with internal cavities.

Multi‐stage casting enables enhanced functionality in soft robotics, as demonstrated by Gong's 3D soft robotic arm (Figure 6d). This process involved the sequential casting of silicone elastomer segments within polylactic acid molds, with rubber tendons integrated to restrict expansion during pneumatic actuation.^[^ ^96^ ^]^ Despite its advantages, multi‐stage casting has drawbacks, including time‐intensive production, ≈10 h for Gong's design, and interfacial weaknesses between materials, increasing the risk of delamination. Potential solutions include process optimization, mechanical interlocking which may limit design flexibility, and specialized techniques such as rotational casting, which is effective for hollow structures but unsuitable for robust multi‐material parts. Advancements in materials and casting technologies are expected to expand robotic applications in demanding environments while addressing current limitations.

### 5.3 Shape Deposition Manufacturing

SDM utilizes a layer‐by‐layer material deposition process to fabricate intricate 3D structures, where each cured layer serves as the foundation for subsequent layers (Figure 6e).^[^ ^93^ ^]^ Unlike conventional 3D printing, SDM excels in multi‐material integration, combining rigid and flexible materials, and in the fabrication of complex geometries such as internal cavities and microfluidic channels without requiring support or extensive post‐processing.^[^ ^97^, ^98^ ^]^ Its precision minimizes residual stress, enhancing durability for applications involving repetitive motion and high loads, such as grippers and actuators.

A key advantage of SDM is its ability to embed sensors and electronic components in situ, enabling fully integrated, multifunctional designs while eliminating the need for post‐assembly. This capability significantly expands its applications in medical, industrial, and exploration robotics.^[^ ^99^ ^]^ Additionally, SDM facilitates localized repair and rapid prototyping, accelerating the production of customized components.

For example, Backus et al. developed a microspine gripper using SDM, embedding a metal hook within a hybrid rigid‐flexible polyurethane holder (Figure 6f). The base flexures allow lateral motion while restricting vertical displacement, while elastic linkages enable load‐sharing across 14 microspines in a cassette, supporting cliff scaling for planetary exploration.^[^ ^100^ ^]^

SDM's ability to fuse multiple materials, create structurally complex designs, and integrate embedded functionalities establishes it as a foundational technique for high‐performance soft robotics in demanding environments.

### 5.4 Soft Lithography

Soft lithography is an advanced micro‐ and nanofabrication technique that utilizes elastic molds such as PDMS to replicate intricate structures with high resolution, cost efficiency, and design flexibility. Widely applied in microfluidics, bioengineering, and soft robotics, it addresses the limitations of traditional methods by reducing equipment costs while supporting diverse applications.^[^ ^88^, ^101^, ^102^ ^]^

Microcontact printing (µCP) uses flexible molds to transfer chemical ink patterns onto substrates, achieving submicron resolution for rapid 2D surface modifications. Despite constraints in structural complexity and mold reusability, µCP remains essential for biosensors and microelectronics.^[^ ^88^ ^]^

Microtransfer molding (µTM) advances 3D fabrication by injecting and curing materials in elastic molds in a single step, as illustrated in Figure 6g.^[^ ^88^ ^]^ While dependent on mold quality and requiring time‐intensive processing, µTM excels in rigid‐flexible hybrid systems. For example, Wehner et al. demonstrated its utility in soft robotics through a microfluidic logic‐controlled robot that autonomously regulated fuel decomposition and pneumatic actuation to facilitate movement.^[^ ^12^ ^]^

Capillary microforming (MIMIC) leverages capillary action to fill microstructures without external pressure, making it well‐suited for precise microfluidic channel fabrication. Although it demands precise mold design and material control, MIMIC provides a cost‐effective solution for deformable actuators in medical and industrial robotics.^[^ ^12^, ^102^ ^]^

Solvent‐assisted micromolding (SAMIM) enables the fabrication of complex geometries and multilayered structures by incorporating solvents, allowing for the processing of high‐viscosity materials. While challenges include solvent selection and evaporation control, SAMIM enhances dynamic deformation capabilities in soft robotics and precision manufacturing for optoelectronic applications.^[^ ^102^ ^]^

Multilayer soft lithography constructs integrated 3D systems by stacking and bonding patterned layers embedded with sensors or fluidic channels. Ranzani et al. showcased its potential through a spider‐inspired robot featuring UV‐cured, fluid‐actuated microchannels for origami‐like folding, highlighting its multifunctional applications, as shown in Figure 6h.^[^ ^102^ ^]^

Each technique serves specific purposes: µCP for rapid 2D patterning, µTM, and SAMIM for 3D and multilayer designs, MIMIC for precision fluidics, and multilayer lithography for functional integration. Future developments will focus on improving precision, expanding material compatibility, and enhancing process efficiency to drive innovations in biomedical, industrial, and exploratory applications.^[^ ^88^, ^101^, ^102^ ^]^

Advancements in soft robotics manufacturing have led to significant improvements in fabrication precision, structural complexity, and multi‐material integration. Techniques such as 3D printing, casting, SDM, and soft lithography offer diverse approaches to constructing soft robots with varying mechanical properties and functional capabilities. Each method presents unique advantages and limitations, requiring careful selection based on the robot's intended application and performance requirements. The focus of soft robotics manufacturing will be on improving scalability, reducing costs, and enhancing material‐process compatibility. Innovations in hybrid fabrication techniques and automated assembly will further enable the development of more adaptable and high‐performance soft robots. Additionally, integrating real‐time sensing and embedded intelligence during fabrication could enhance robotic functionality, leading to more autonomous and responsive systems capable of operating in dynamic environments.

Advances in soft robotics manufacturing have improved fabrication precision, structural complexity, and multi‐material integration. Techniques such as 3D printing, casting, SDM, and soft lithography enable customized mechanical properties and functionalities, each offering unique advantages and limitations that must align with specific application requirements. Future developments will focus on scalability, cost efficiency, and material‐process compatibility, driven by innovations in hybrid fabrication and automated assembly. The integration of real‐time sensing and embedded intelligence during production could enhance robotic autonomy, enabling adaptive systems for dynamic environments.

## 6 Control Strategies of Soft Robotics

**Figure 7:**
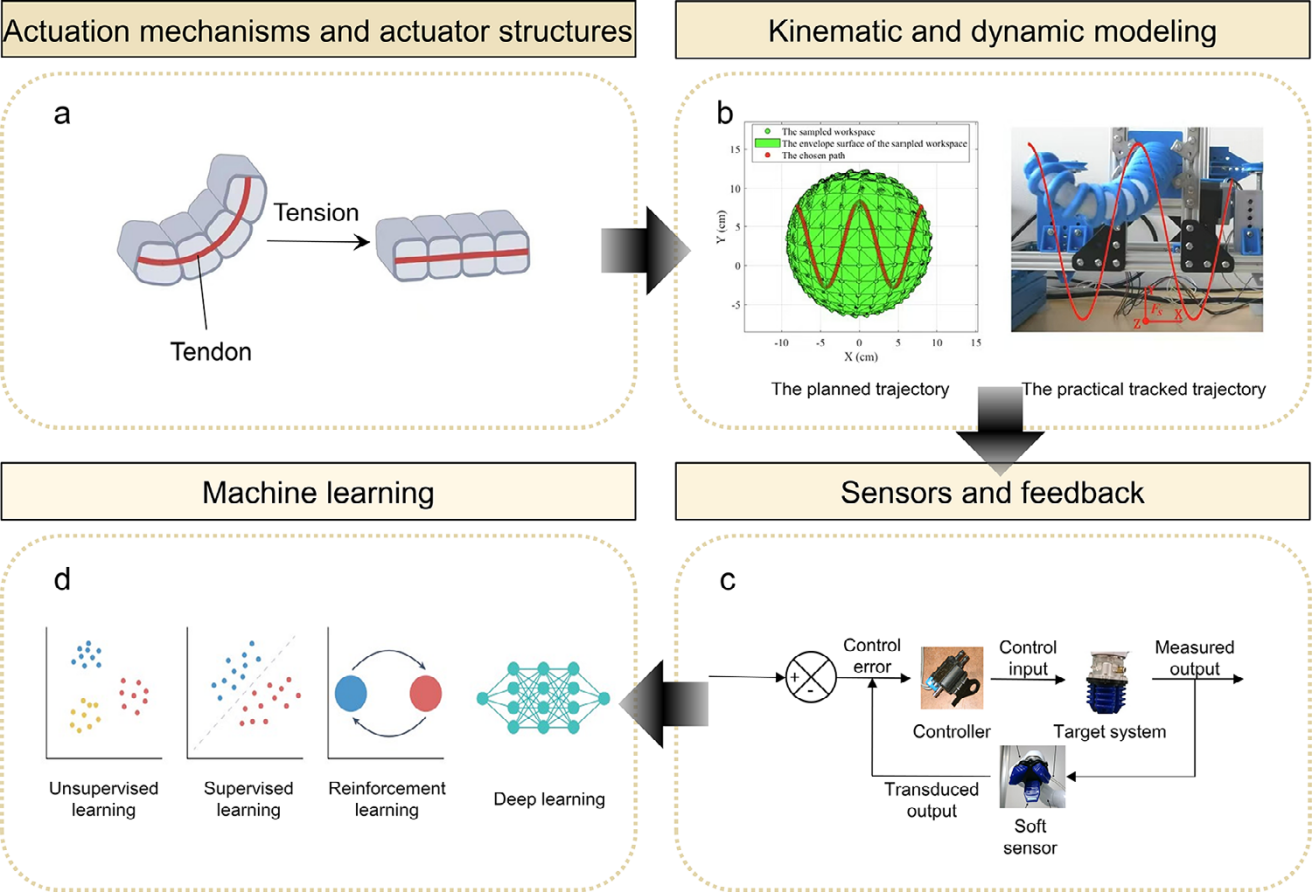
Schematic illustration of soft robot control strategies. a) Control strategies based on actuation mechanisms and actuator structures (tendon‐driven schematic).^[^ ^1^ ^]^ Reproduced with permission.^[^ ^1^ ^]^ Copyright 2024, Springer Nature. b) Control strategies based on kinematics and dynamics modeling, exemplified by a soft elephant trunk robot using finite element method combined with model predictive control for dynamic control, tested for the feasibility and robustness of a time‐constrained 3D sinusoidal trajectory.^[^ ^107^ ^]^ Reproduced with permission.^[^ ^107^ ^]^ Copyright 2022, Elsevier. c) Control strategies based on sensor feedback (closed‐loop feedback mechanism schematic).^[^ ^111^ ^]^ Reproduced with permission.^[^ ^111^ ^]^ Copyright 2021, John Wiley & Sons. d) Control strategies based on machine learning (schematic of unsupervised learning, supervised learning, reinforcement learning, and deep learning).^[^ ^1^ ^]^ Reproduced with permission.^[^ ^1^ ^]^ Copyright 2024, Springer Nature.

The effective selection of control strategies significantly enhances soft robots' performance, task adaptability, and real‐world deployment.^[^ ^104^ ^]^ These strategies are categorized into frameworks based on actuation mechanisms,^[^ ^105^, ^106^ ^]^ such as pneumatic, hydraulic, and tendon‐driven systems, which ensure precise deformation and motion efficiency through mechanical alignment. Over time, advancements in actuation mechanisms have led to the integration of more adaptive and responsive control strategies, transitioning from purely mechanical models to hybrid approaches incorporating real‐time feedback and computational intelligence. Kinematic and dynamic modeling provides theoretical foundations for optimizing complex behaviors and trajectory planning,^[^ ^107^, ^108^, ^109^ ^]^ while sensing and feedback mechanisms^[^ ^110^, ^111^, ^112^, ^113^ ^]^ leverage real‐time data processing and bio‐inspired receptors to improve environmental adaptability and precision. Additionally, machine learning enhances autonomy by enabling data‐driven decision‐making and predictive adaptability for intelligent task execution.^[^ ^114^, ^115^ ^]^ This evolution from conventional rule‐based control to AI‐driven frameworks has significantly improved soft robots' ability to interact dynamically with uncertain environments. Collectively, these methods (as shown in **Figure** **7**) drive advancements in biomedical, industrial, and bio‐assistive applications, accelerating functional innovation and adoption.

### 6.1 Control Strategies Based on Actuation Mechanisms and Actuator Structures

The actuation mechanisms of soft robotics fundamentally influence control strategy design by determining implementation approaches and control complexity. Actuation mechanisms—including pneumatic, hydraulic, SMA, DE, and magnetic systems—exhibit distinct dynamic responses and control requirements. Pneumatic actuators necessitate precise pressure‐flow regulation to compensate for inflation and deflation delays, while SMAs require thermal nonlinearity management. Therefore, control strategies must balance speed, precision, and stability based on the dynamics of each actuation mechanism.^[^ ^106^ ^]^

Actuator flexibility, multi‐degree‐of‐freedom motion, and distributed properties further shape control system design. Tendon‐driven systems require tension‐displacement regulation for precise motion,^[^ ^106^ ^]^ whereas distributed pneumatic chambers demand zonal coordination. This necessitates strategies such as closed‐loop feedback, distributed control, and multi‐modal algorithms to ensure structural synergy. Such systems exemplify bio‐inspired actuation, utilizing cable tension to facilitate bending and stretching through motors or pneumatic units (Figure 7a).^[^ ^1^ ^]^ However, challenges include nonlinear dynamics, hysteresis due to tendon friction and elongation, and multi‐degree‐of‐freedom coordination. Real‐time sensor feedback and distributed control algorithms help mitigate these issues. For example, Gunderman et al.^[^ ^105^ ^]^ developed a tendon‐driven gripper with force feedback and a closed‐loop control strategy to maintain consistent grip force when handling fragile objects. This design leverages passive compliance for delicate tasks while addressing hysteresis through intelligent feedback integration. Despite these advancements, nonlinear behaviors remain a key limitation.

Actuation mechanisms and architectures dictate control strategy selection based on their inherent dynamic properties. Integrating bio‐inspired design, material compliance, and feedback mechanisms can address nonlinear control challenges in soft robotics. Future advancements require intelligent algorithms, real‐time sensing, and efficient modeling tools to enhance precision and stability for practical applications.

### 6.2 Control Strategies Based on Kinematic and Dynamic Modeling

The kinematic and dynamic modeling of soft robotics is fundamental to developing effective control strategies, as it determines accuracy and feasibility. Kinematic models establish the relationship between input signals, such as actuation forces, and the resulting deformation and posture changes, facilitating path planning and target positioning. Dynamic models extend this by incorporating external and internal forces, inertia, and damping, revealing the input‐behavior relationships during motion.^[^ ^108^ ^]^ These models are essential for predicting complex system responses.

Kinematic modeling is primarily used for path planning and static deformation control, such as inverse kinematics for posture adjustment, whereas dynamic modeling focuses on force regulation and motion optimization in dynamic scenarios, including object interactions and stability‐speed balancing.^[^ ^109^ ^]^ However, the nonlinear and flexible structures of soft robots pose modeling challenges, often requiring sensor feedback and optimization algorithms to ensure robust real‐time control.

Finite element analysis (FEA) plays a crucial role in simulating deformation, mechanical responses, and control optimization. By discretizing structures into elements and solving stress‐strain relationships, FEA predicts actuation‐induced motion and refines control parameters to enhance accuracy. It also validates control algorithms experimentally, reducing the need for extensive iterative testing in nonlinear systems. For example, Wu et al.^[^ ^107^ ^]^ developed an FEA‐based dynamic controller for a soft trunk‐like robot, integrating model predictive control with a time‐constrained 3D trajectory (Figure 7b). Experimental results confirmed strong trajectory‐tracking precision, feasibility, and robustness against disturbances, highlighting FEA's critical role in dynamic soft robot control.

Although kinematic and dynamic models enable quantitative analysis and prediction, challenges remain in managing nonlinearities, parameter identification, and computational demands. Future advancements in real‐time feedback integration and intelligent algorithms will improve adaptability and precision, driving the application of soft robotics in complex and dynamic environments.

### 6.3 Control Strategies Based on Sensors and Feedback

The sensor and feedback systems in soft robotics are essential for precise control and closed‐loop regulation, enabling real‐time monitoring of parameters such as deformation, pressure, and position. This data facilitates dynamic input adjustments, ensuring stability and adaptability in unpredictable environments. Additionally, these systems mitigate nonlinearities and coupling effects in multi‐degree‐of‐freedom structures, improving coordination and efficiency.^[^ ^110^, ^112^ ^]^

Soft robot control strategies are generally classified into open‐loop systems, which rely on pre‐programmed inputs without feedback, and closed‐loop systems, which use real‐time feedback for dynamic adjustments. Hybrid approaches integrate open‐loop execution with closed‐loop corrections, proving effective in nonlinear systems or environments with disturbances. Common feedback mechanisms include visual, force, and tactile sensing.^[^ ^104^, ^113^ ^]^

For example, Sekine et al. developed a ferroelectric polymer‐nanocarbon soft sensor with enhanced crystallinity through annealing, achieving a polarization of 11.0 µC cm^−^^2^ and superior shear force detection. When integrated into a gripper with closed‐loop feedback, the sensor‐enabled rapid slip detection, allowing for automatic grip adjustments in response to slipping objects. This demonstrated adaptive task execution without requiring additional training, highlighting its potential for bio‐inspired electronic skin applications (Figure 7c).^[^ ^111^ ^]^

Sensor‐driven feedback enhances precision in pneumatic arms and grippers, driving advancements in medical rehabilitation and human‐robot interaction. However, challenges such as sensor density, data processing efficiency, and latency remain significant. Future research should focus on optimizing receptor‐inspired sensor integration, minimizing latency, and refining fusion algorithms to fully harness the potential of soft robots in complex real‐world applications.

### 6.4 Control Strategies Based on Machine Learning

Machine learning (ML), encompassing unsupervised, supervised, reinforcement, and deep learning (Figure 7d),^[^ ^1^ ^]^ provides data‐driven solutions for soft robotics by addressing system nonlinearity, structural complexity, and environmental uncertainty. Unsupervised learning identifies patterns in unlabeled data, while supervised learning relies on labeled datasets for predictive tasks such as motion trajectory planning. Reinforcement learning optimizes control policies through environmental interaction, making it particularly effective for dynamic scenarios like adaptive gripper force control. Deep learning utilizes neural networks to process multisensory data, enabling precise state estimation and decision‐making for complex tasks such as pneumatic arm pressure regulation.^[^ ^115^ ^]^

A notable application by Sun et al. integrates dual hydrogel sensors with a hybrid ML model combining 1D convolutional neural network and feed‐forward neural network for multimodal soft robot perception. The system achieved 86.3% accuracy in real‐time recognition of bending, twisting, thermal contact, and mechanical stimuli, demonstrating reliable proprioceptive and environmental sensing for intelligent actuation control.^[^ ^114^ ^]^

ML‐based control enhances adaptability in unstructured environments where traditional physics‐based models face limitations. However, challenges remain in data acquisition efficiency, real‐time processing, and model generalization. Future hybrid frameworks that integrate data‐driven learning with physics‐informed models hold promise for improved precision and stability, advancing soft robotics applications in medical, industrial, and bio‐inspired domains.

In addition to the previously mentioned control strategies, the rapid development of neuromorphic electronics has introduced innovative approaches to the control of soft robots. Researchers have combined bio‐inspired artificial synapses^[^ ^116^, ^117^, ^118^, ^119^, ^120^, ^121^ ^]^ with flexible biomaterials to develop neuromorphic computing chips with learning and control capabilities, laying a critical foundation for neural‐like control in soft robots. Meanwhile, recent advances in flexible memristors,^[^ ^122^, ^123^, ^124^ ^]^ particularly in advanced computing and sensing applications, have further enhanced the information processing and environmental sensing capabilities of soft robots. Moreover, emerging intelligent flexible sensing systems driven by machine learning and artificial synapses have not only significantly improved the sensory accuracy of soft robots but also accelerated the development of data‐driven adaptive control strategies.^[^ ^126^, ^127^, ^128^ ^]^ Overall, these technological breakthroughs provide robust support for soft robots to achieve efficient perception and adaptive regulation in complex environments, enabling them to leverage neural network‐like architectures for real‐time sensing and self‐adaptive adjustment, marking a key step toward greater intelligence and biomimetics.

Beyond conventional control strategies, neuromorphic electronics provide transformative strategies for soft robotics. The integration of bio‐inspired artificial synapses^[^ ^116^, ^117^, ^118^, ^119^, ^120^, ^121^ ^]^ with flexible biomaterials enables neuromorphic chips, establishing neural‐like control foundations. Advances in flexible memristors^[^ ^122^, ^123^, ^124^, ^125^ ^]^ enhance computational efficiency and environmental sensing capabilities, while machine learning‐driven intelligent sensing systems improve sensory precision and accelerate adaptive control.^[^ ^126^, ^127^, ^128^ ^]^ These innovations equip soft robots with real‐time perception and self‐regulation through neural network‐inspired architectures, marking significant progress toward bioinspired intelligence in complex environments.

## 7 Emerging Applications for Soft Robotics

Soft robotics have shown broad potential across various application areas, including biomimetics, navigational movement, grasping operations, biomedical devices, and wearable robots. In the field of biomimetics, soft robots mimic the form and motion mechanisms of biological organisms, exhibiting functions similar to biological systems, which allows them to adapt to complex environments and perform delicate tasks. In navigational movement, soft robots use bioinspired movement patterns to complete search and rescue missions in confined spaces, showcasing unique spatial adaptability. In grasping operations, soft robots rely on their soft and deformable structures to safely handle fragile objects, making them suitable for precision industrial work and medical applications. In biomedical devices, soft robots are widely used in minimally invasive surgeries, flexible endoscopes, and rehabilitation treatments, helping to improve medical precision while enhancing patient comfort. Additionally, wearable robots provide dynamic support for people with mobility impairments and help reduce labor intensity in industrial production, improving work efficiency. These advancements provide innovative solutions for both scientific studies and engineering applications.

### 7.1 Soft Sensors

**Figure 8:**
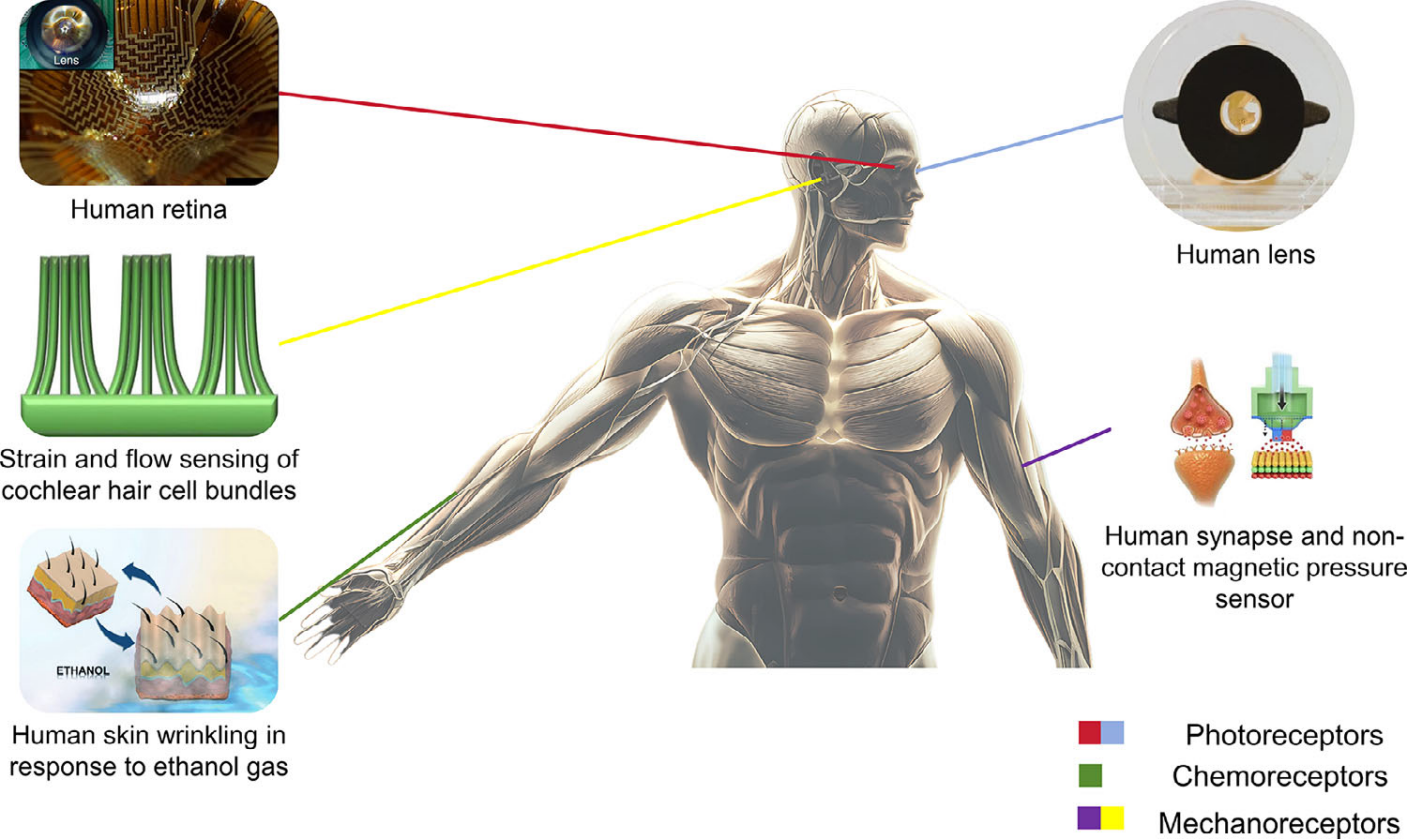
Soft sensors inspired by human receptors, including photoreceptors (simulating the human lens^[^ ^133^ ^]^ and retina^[^ ^131^ ^]^), chemoreceptors (mimicking the cold sensation, slight stinging, or wrinkling reaction when skin comes into contact with ethanol gas^[^ ^135^ ^]^), and mechanoreceptors (inspired by the sensitivity of cochlear hair cell bundles to fluid flow caused by sound waves,^[^ ^138^ ^]^ leading to the design of flow sensors, as well as non‐contact magnetic pressure sensors inspired by the non‐contact signal transmission mechanism in human synapses^[^ ^139^ ^]^). Reproduced with permission.^[^ ^133^ ^]^ Copyright 2015, John Wiley & Sons. Reproduced with permission.^[^ ^131^ ^]^ Copyright 2017, Springer Nature. Reproduced with permission.^[^ ^135^ ^]^ Copyright 2019, John Wiley & Sons. Reproduced with permission.^[^ ^138^ ^]^ Copyright 2019, Springer Nature. Reproduced with permission.^[^ ^139^ ^]^ Copyright 2017, Springer Nature.

Soft sensors also play a crucial role in the development of soft robots. They can monitor key parameters such as deformation, pressure, and temperature in real‐time, providing precise environmental sensing and feedback capabilities. This helps the robot dynamically adjust its behavior and ensures efficient interaction with its environment.^[^ ^129^, ^130^ ^]^ The application of soft sensors significantly enhances the adaptability and intelligence of soft robots, offering strong technical support for their widespread application in various fields. The design and development of soft sensors are profoundly inspired by biological systems, particularly the sensitive perception mechanisms of various human receptors. These bioinspired mechanisms provide soft robots with advanced sensing capabilities, enabling them to achieve highly accurate perception and feedback even in complex environments. Human receptors can be broadly categorized into three main types: photoreceptors, chemoreceptors, and mechanoreceptors, as illustrated in **Figure** **8**. Each type of receptor possesses unique sensing functions, offering invaluable theoretical foundations and references for the bioinspired design of soft sensors.^[^ ^7^ ^]^

#### 7.1.1 Photoreceptors

Photoreceptors are a key component of the visual system, responsible for sensing light signals and converting them into electrical signals, enabling the recognition of light intensity, color, and shape. In recent years, innovations based on origami technology, semiconductor nanomembranes, and soft materials have advanced the development of bio‐inspired photoreceptors. For example, by combining origami folding with nanomembrane technology, researchers have successfully created high‐performance, compact, and easily scalable photodetector arrays, significantly enhancing soft robots' visual navigation and environmental perception capabilities.^[^ ^131^ ^]^ A fully printed, stretchable corneal sensor based on soft contact lenses, made using DIW technology, has overcome the limitations of traditional rigid corneal sensors, with potential for mass production and widespread application in ophthalmic diagnostics and monitoring.^[^ ^132^ ^]^

In the field of soft photonic sensors, a flexible, tunable‐focus lens mimicking the human eye's lens has been developed using elastomeric materials. This lens enables rapid focal adjustments and dynamic visual perception, making it particularly suitable for optical navigation and environmental monitoring. With high responsiveness, low energy consumption, and excellent stability, this lens is expected to become a core component of soft robot vision systems.^[^ ^133^ ^]^ The development of artificial photoreceptors expands the application potential of soft robots in visual perception and navigation while providing key support for the advancement of bio‐inspired visual systems, with broad prospects for use in medical diagnostics, environmental monitoring, and intelligent robotics.

Additionally, recent discoveries in neuroscience have revealed that non‐traditional photoreceptors, such as intrinsically photosensitive retinal ganglion cells, play a significant role in enhancing visual orientation processing in mammals by modulating cortical excitation and inhibition.^[^ ^134^ ^]^ Although not yet applied to soft robotics, this newly uncovered visual pathway suggests novel directions for designing artificial photoreceptors with multisensory modulation capabilities in future bioinspired robotic vision systems.

#### 7.1.2 Chemoreceptors

Chemoreceptors are key receptors in the human body responsible for detecting chemical stimuli, primarily located in the olfactory, gustatory systems, and skin. They quickly respond to chemical changes in the environment. Inspired by these bioinspired mechanisms, bioinspired chemical sensors are capable of real‐time monitoring of gas, liquid, or specific molecule concentrations in the environment and are widely used in pollution detection, gas leak monitoring, and medical diagnostics, providing soft robots with strong environmental chemical sensing and adaptive response capabilities.

For example, a flexible sensor based on AgNW/SiO_x_/PDMS multilayer films utilizes a dynamic surface wrinkling mechanism to respond to volatile organic compounds, demonstrating high selectivity and sensitivity, suitable for real‐time environmental monitoring.^[^ ^135^ ^]^ Additionally, a mineral hydrogel‐based sensor employs a capacitance imbalance mechanism to precisely measure the mass and angle of water droplets, offering self‐healing properties and high sensitivity.^[^ ^136^ ^]^ These sensors not only improve the efficiency of environmental monitoring but also provide soft robots with precise chemical sensing capabilities, enabling them to perform tasks such as detection and recognition in complex environments.

In a recent study, researchers developed a magnetically actuated liquid‐phase soft robot based on a coacervate formed from interactive magnetic nanoparticles. The system integrates biomarker‐responsive degradation with active transport, enabling the robot to chemically sense its surroundings and trigger targeted drug release. Its high deformability and fluidic body allow it to traverse narrow environments, while its release function is activated by specific chemical cues. This represents a step toward chemoresponsive soft robotic systems capable of autonomous adaptation, navigation, and task execution in chemically dynamic environments.^[^ ^137^ ^]^

#### 7.1.3 Mechanoreceptors

Mechanoreceptors are widely distributed in the human skin, joints, and cochlea, and are responsible for sensing mechanical stimuli such as pressure, touch, vibration, and deformation. For example, the hair cells in the cochlea can detect small deformations and flow changes, providing inspiration for flow sensor designs; the fast‐adapting receptors in the skin, such as Meissner's corpuscles, respond to light touch and dynamic vibrations, while slow‐adapting receptors, like Merkel's discs, continuously sense static pressure and deformation. These biological mechanisms serve as the foundation for the design of bioinspired mechanosensors, which are widely used in fields such as flexible grippers, bioinspired electronic skin, and flow detection systems, enhancing the performance of soft robots in flexible manipulation, deformation control, and adaptability to complex environments.

Miao et al. were inspired by the hair cells in the cochlea and designed a nanowire‐assembled sensor that utilizes the crack generation mechanism in micro and nano pores, achieving ultra‐high stretchability and high sensitivity.^[^ ^138^ ^]^ This design offers excellent flexible sensing solutions for wearable devices and soft robots. Oh et al. developed a remote tactile sensor based on a bioinspired synaptic system, which converts external pressure into an electrical signal through changes in air pressure, successfully enhancing the sensitivity and dynamic range of tactile perception, and is applied in surface texture recognition and underwater sensing.^[^ ^139^ ^]^ Huynh et al. proposed a bioinspired sensor combining self‐powered piezoelectric pressure sensors and electrolyte‐gated field‐effect transistors, successfully simulating the tactile perception functions of fast‐adapting and slow‐adapting receptors, providing new technical support for smart electronic skin and artificial neural robots.^[^ ^140^ ^]^

As described above, photoreceptors, chemoreceptors, and mechanoreceptors provide biological inspiration for the development of soft sensors, endowing soft robots with multimodal sensing capabilities. These receptor‐inspired sensors not only enable high‐sensitivity detection of optical, chemical, and mechanical signals, but also enhance the system's adaptability and accuracy through intelligent feedback mechanisms. For example, the focusing mechanism of the lens inspired the design of adaptive optical sensors, the skin's response to chemical substances advanced the development of chemosensing technology, and the cochlear hair cell bundle and non‐contact magnetic pressure sensors enable precise tactile and flow sensing. These receptor‐inspired sensors work together to significantly improve the adaptability and performance of soft robots in complex environments.

In a recent study, researchers developed bioinspired artificial synaptic mechanoreceptors that integrate triboelectric gating and synaptic transistors into a vertical tactile architecture. By mimicking both slowly adapting and fast‐adapting afferent neuron behavior, these devices generate excitatory post‐synaptic current (EPSC) signals with tactile memory and adaptability. When arranged in arrays, the device enabled intelligent electronic skin to distinguish handwriting styles, textures, and surface patterns using machine learning, while reducing data redundancy and energy consumption. This synapse‐like mechanosensory architecture offers promising prospects for edge‐AI‐enabled soft robotic systems.^[^ ^141^ ^]^

### 7.2 Biomimetics

**Figure 9:**
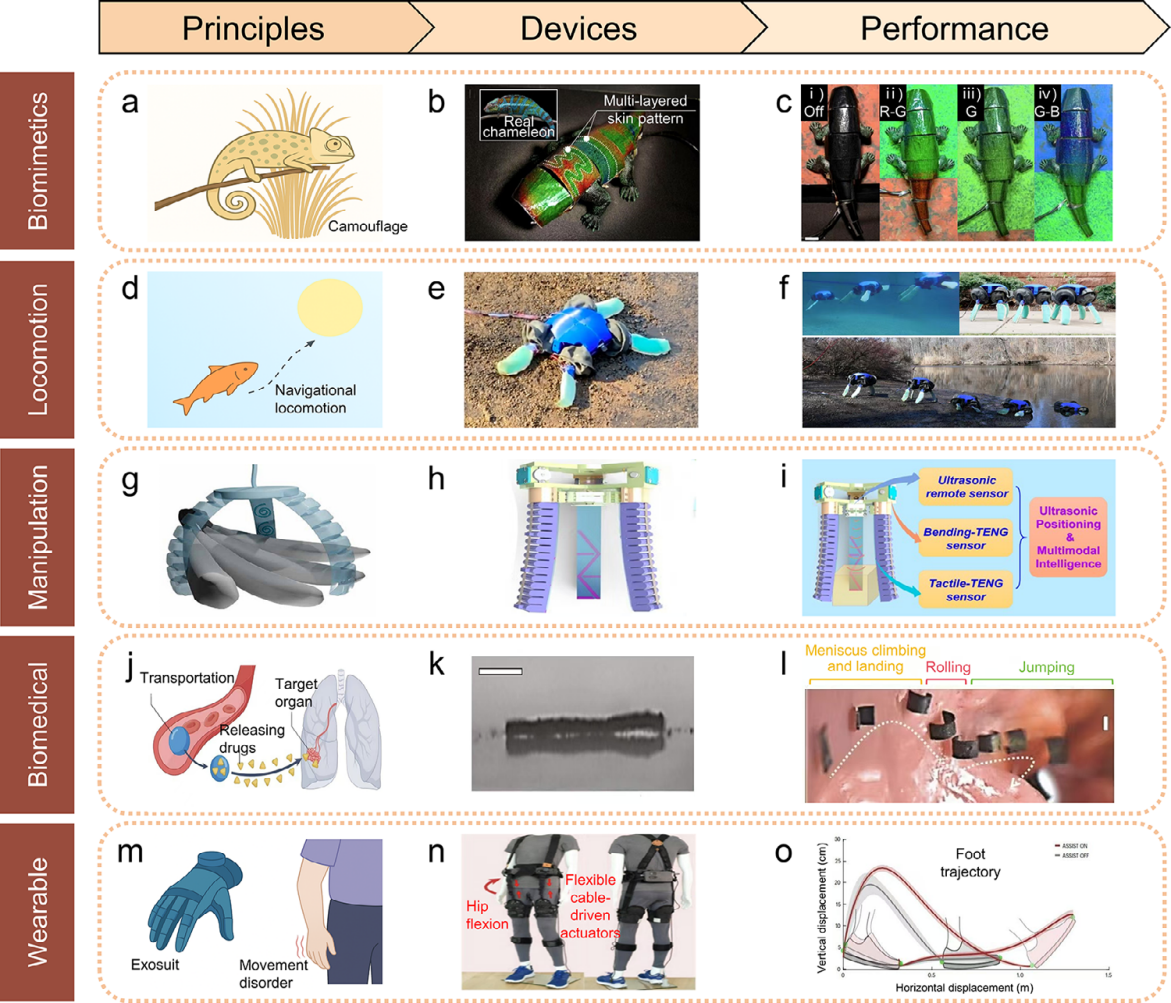
Typical application scenarios of soft robots. a) Illustration of the bionic field (camouflage and concealment). b) Artificial camouflage technology based on thermochromic liquid crystal layers and vertically stacked silver nanowire heaters.^[^ ^145^ ^]^ c) A chameleon‐inspired soft robot capable of detecting and matching local environmental colors in real time, naturally transitioning to display specific patterns.^[^ ^145^ ^]^ Reproduced with permission.^[^ ^145^ ^]^ Copyright 2021, Springer Nature. d) Illustration of environmental exploration and navigation. e) Image of an amphibious bionic robotic turtle.^[^ ^146^ ^]^ f) ART robots capable of efficient movement on land, underwater, and in transitional zones.^[^ ^146^ ^]^ Reproduced with permission.^[^ ^146^ ^]^ Copyright 2022, Springer Nature. g) Illustration of the flexible gripper field.^[^ ^11^ ^]^ Reproduced with permission.^[^ ^11^ ^]^ Copyright 2020, AAAS. h) A flexible gripper integrating ultrasonic automatic positioning and multimodal intelligent sensing.^[^ ^155^ ^]^ i) By combining ultrasonic sensors and flexible triboelectric sensors, this gripper can remotely locate objects and precisely sense multimodal data such as shape, size, hardness, and material.^[^ ^155^ ^]^ Reproduced with permission.^[^ ^155^ ^]^ Copyright 2023, American Chemical Society. j) Illustration of the biomedical technology field (minimally invasive surgery and drug delivery).^[^ ^1^ ^]^ Reproduced with permission.^[^ ^1^ ^]^ Copyright 2024, Springer Nature. k) Illustration of a wireless magnetoelastic millimeter‐scale soft robot.^[^ ^157^ ^]^ l) This robot, driven by external magnetic forces, demonstrates excellent multimodal motion capabilities, including swimming in and out of liquids, climbing, rolling, overcoming obstacles, and crawling through narrow passages.^[^ ^157^ ^]^ Reproduced with permission.^[^ ^157^ ^]^ Copyright 2018, Springer Nature. m) Illustration of the field of rehabilitation and smart wearable technologies. n) Flexible robotic clothing designed to mitigate freezing of gait in Parkinson's patients.^[^ ^169^ ^]^ o) By assisting hip flexion, it effectively improves freezing of gait, significantly enhancing patients’ walking ability, gait quality, and independence.^[^ ^169^ ^]^ Reproduced with permission.^[^ ^169^ ^]^ Copyright 2024, Springer Nature.

Soft robots have made significant progress in bioinspired stealth and camouflage technologies, drawing inspiration from the characteristics of organisms such as jellyfish, transparent insects, chameleons, and octopuses, which enhances their ability to blend into the environment and expand functionality (**Figure** **9**a).^[^ ^142^, ^143^ ^]^ In terms of stealth technology, researchers have developed transparent soft robots integrated with transparent electronic components, enabling them to maintain visual concealment while performing tasks, minimizing disruption to the surrounding environment. Regarding camouflage technology, Wang et al. combined stretchable films with nanostructures to develop a bioinspired chameleon capable of quickly responding to color changes and seamlessly blending with the background.^[^ ^144^ ^]^ Additionally, Kim et al. developed camouflage technology based on thermochromic liquid crystal layers and AgNW heaters (Figure 9b), enabling soft robots to match the surrounding environment's color in real‐time, improving camouflage effect and stability, and demonstrating potential applications in fields such as military and architecture (Figure 9c).^[^ ^145^ ^]^

### 7.3 Locomotion

Soft robots demonstrate broad application potential in the field of locomotion, particularly in environmental exploration and navigation applications (Figure 9d). Thanks to their flexible materials and deformable structures, soft robots can adapt flexibly to complex terrains, overcoming the limitations of traditional rigid robots in unstructured environments. In environmental exploration, soft robots mimic the movement modes of various organisms, such as crawling and swimming, enabling them to access areas that are difficult for humans to reach for data collection and monitoring. The adaptive morphogenesis design proposed by Baines et al. (Figure 9e) allows robots to move efficiently on both land and underwater while enhancing adaptability through flexible gait changes.^[^ ^146^ ^]^ A notable real‐world example is the soft robotic gripper developed by Harvard researchers, which was deployed at depths of over 800 m in the Red Sea to collect fragile coral specimens.^[^ ^147^ ^]^ Faced with challenges such as high hydrostatic pressure and delicate biological structures, the team employed soft silicone actuators and pressure‐tolerant control systems. This compliant design enabled precise, non‐destructive grasping in extreme marine environments, showcasing the advantages of soft robotics in sensitive deep‐sea exploration tasks. In navigation, soft robots integrate intelligent control and embedded sensors to achieve autonomous navigation in complex environments. They can avoid obstacles and dynamically adjust their paths, making them suitable for search and rescue tasks. For example, Zhao et al.’s robot based on liquid crystal elastomers (Figure 9f) utilizes material intelligence and geometric asymmetry to autonomously adjust its path in complex environments like mazes, demonstrating excellent navigation capabilities.^[^ ^148^ ^]^

### 7.4 Manipulation

Soft robots have shown some potential in the field of manipulation, particularly in the application of flexible grippers (Figure 9g).^[^ ^11^, ^149^, ^170^ ^]^ These soft grippers use flexible, deformable materials to interact safely with objects of varying shapes, materials, and fragility, thus expanding the scope and flexibility of manipulation tasks. For example, the gripper developed by Shintake et al., based on DEA, can efficiently grasp objects such as paper, eggs, and balloon films, with features like fast response and high holding force.^[^ ^150^ ^]^ Additionally, grippers integrated with soft sensors can monitor the gripping process in real‐time, dynamically adjusting the force to prevent object slippage or damage, thereby improving the precision and safety of manipulation.

To enhance intelligence, machine learning techniques have been incorporated into the operating system, providing the grippers with stronger data analysis and decision‐making capabilities. By training algorithms, soft grippers can accurately identify the type, location, and state of objects, optimizing the gripping strategy. The combination of deep learning and multimodal sensors has significantly improved the cognitive ability and adaptability of soft robots in complex environments.^[^ ^151^, ^152^, ^153^, ^154^ ^]^ In particular, the soft robot system proposed by Shi et al. (Figure 9h), which integrates ultrasonic automatic localization and multimodal intelligent sensing (Figure 9i), can accurately identify objects and adjust the strategy in real‐time during manipulation, significantly improving the recognition accuracy and stability of the system.^[^ ^155^ ^]^

### 7.5 Biomedical Devices

Soft robots in biomedical applications are primarily used in minimally invasive surgery and drug delivery (Figure 9j). Their flexibility and lightweight characteristics allow them to match well with human tissue, reducing mechanical damage and inflammation. Theoretical principles such as mechanical compliance, low Young's modulus, and bioinspired morphing allow soft robots to conform to dynamic, irregular anatomical structures—such as convoluted blood vessels or delicate neural networks—thereby minimizing invasive force and enhancing safety. In minimally invasive surgery, soft robots, with their flexible actuators and deformable structures, can adapt to complex anatomical pathways such as blood vessels and the brain, improving surgical precision and success rates.^[^ ^156^ ^]^ For instance, magnetically actuated or dielectric elastomer‐based soft arms allow for programmable curvature and force modulation, enabling surgeons to access narrow spaces without damaging surrounding tissues. The cable‐free magnetoelastic soft robot designed by Hu et al. (Figure 9k) demonstrates exceptional multi‐mode mobility (Figure 9l), capable of switching freely between liquid and solid terrains and performing various tasks.^[^ ^157^ ^]^

In drug delivery, soft robots use external stimuli, such as magnetic fields and ultrasound, to precisely control their path and achieve targeted drug release, enhancing treatment efficiency and reducing side effects. Their soft material composition, often involving hydrogel or biodegradable polymer composites, ensures biocompatibility and allows controlled diffusion or mechanical release of therapeutic agents. For example, the bioresorbable acoustic microrobot developed by Han et al., which integrates magnetic nanoparticles and drugs, can stably operate in biological fluids and deliver drugs precisely, showing potential in minimally invasive surgery and disease diagnosis.^[^ ^158^ ^]^

Micro‐ and nanorobots demonstrate unique advantages in the biomedical field, with their extremely small size and high‐precision operational capabilities being particularly remarkable.^[^ ^159^ ^]^ At these scales, physical actuation principles such as magnetically induced torque or localized thermal deformation are essential for precise manipulation in confined biological environments. When integrated with machine learning, their ability to analyze complex biological data in real time is further enhanced, making them highly promising for applications such as targeted drug delivery, lesion localization, tissue repair, and minimally invasive surgery.^[^ ^160^ ^]^ However, despite significant progress in this area, achieving greater stability and broader applicability in real‐world scenarios remains a critical challenge to address.

### 7.6 Wearable Robots

The application of soft robots in wearable devices is primarily reflected in rehabilitation therapy and smart wearable technology (Figure 9m). In the field of rehabilitation, soft robots provide personalized support for patients with motor disabilities through exoskeleton devices and wearable braces.^[^ ^4^ ^]^ These devices are suitable for multiple body parts and can offer customized rehabilitation training programs through intelligent systems.^[^ ^161^, ^162^, ^163^, ^164^ ^]^ For example, the Myoshirt device uses textile materials to provide anti‐gravity support for the shoulder, extend muscle endurance, and reduce muscle activity.^[^ ^165^ ^]^ Additionally, by integrating remote rehabilitation technology, soft robotic devices are gradually transitioning from hospitals to home use.^[^ ^161^ ^]^ In the field of smart wearable technology, these devices are typically equipped with advanced sensors and machine learning algorithms, enabling real‐time monitoring of the user's movement, muscle activity, etc., and dynamically adjusting output forces to achieve more natural motion assistance.^[^ ^166^, ^167^, ^168^ ^]^ Soft robots also demonstrate significant potential in addressing specific medical issues. For instance, freezing of gait (FoG) is a common and severe gait disorder in Parkinson's disease patients. To address this, flexible robotic garments (Figure 9n) can effectively assist with hip flexion, eliminate gait freezing, and significantly improve the patient's gait quality and independence (Figure 9o), opening up new technological avenues for the treatment of FoG.^[^ ^169^ ^]^

The diverse applications of soft robotics demonstrate their potential to revolutionize biomedical engineering, environmental exploration, industrial automation, and human‐machine interfaces. By leveraging bioinspired actuation mechanisms, programmable materials, multiscale manufacturing techniques, and intelligent control strategies, soft robots overcome traditional limitations in adaptability, precision, and human interaction. Their compliant structures and sensory integration enable them to function effectively in complex and dynamic environments, performing tasks ranging from delicate surgical procedures to autonomous environmental monitoring. The continued integration of artificial intelligence, sensor fusion, and multifunctional materials will drive further advancements in soft robotics adaptability, autonomy, and efficiency. Future research should focus on enhancing durability, real‐time responsiveness, and control precision, ensuring that soft robots can operate reliably in increasingly demanding scenarios. As soft robotics technology evolves, it is expected to play an even more prominent role in healthcare, industry, and exploration, redefining how robots interact with the world and expanding their capabilities across a broad spectrum of applications.

## 8 Conclusion and Perspectives

**Figure 10:**
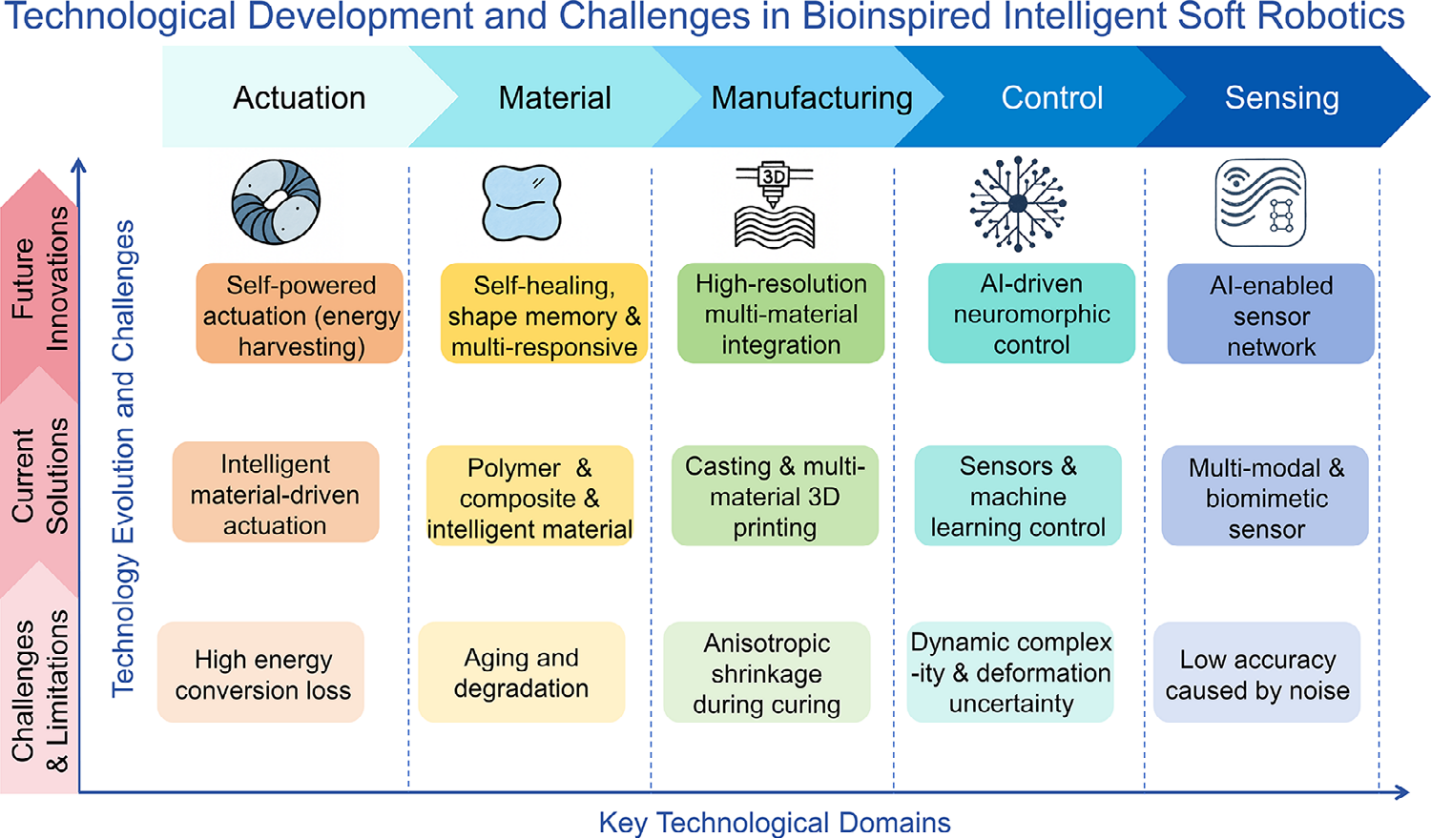
Technological advancements and challenges in bioinspired intelligent soft robotics. The vertical axis shows progress from current limitations to future innovations across five key domains: Actuation, Material, Manufacturing, Control, and Sensing. Each domain evolves from fundamental issues such as energy loss, aging, and low accuracy to advanced solutions such as self‐powered actuation, AI‐enabled sensing, and high‐resolution manufacturing. This roadmap outlines the progression from fundamental limitations to cutting‐edge innovations across five domains, providing a structured comparison to support strategic design in next‐generation soft robotics.

Soft robotics, representing an emerging paradigm in intelligent system engineering, embodies a captivating convergence of multidisciplinary integration, such as actuator design, materials science, manufacturing processes, control algorithms, and sensor technologies. Its applications have proliferated across diverse sectors, notably in healthcare innovations, industrial automation systems, environmental exploration technologies, and next‐generation wearable devices. Significant progress has been made in medical rehabilitation devices, bioinspired artificial muscles, and adaptive robotic grippers for complex manipulation. These achievements have laid a strong technical foundation for developing highly flexible, intelligent, and multifunctional soft robots. **Figure** **10** outlines the technological evolution and challenges in bioinspired intelligent soft robotics, tracing the journey from fundamental limitations to cutting‐edge innovations across five domains across five key domains encompassing actuation, materials, control, manufacturing, and sensing.

Despite significant advancements, soft robotics encounters notable technological barriers. Actuation systems grapple with high energy conversion loss, hindering the attainment of high power density and efficient multi‐degree‐of‐freedom motion. Materials that suffer from time‐dependent degradation such as fatigue cracking, hydrolysis, and UV‐induced embrittlement, pose durability and environmental robustness challenges for long‐term applications. Manufacturing techniques face challenges such as micron‐level tolerance discrepancies, anisotropic shrinkage during curing, and limited repeatability, impacting scalability and unit cost. The control of soft robots is hindered by their dynamic complexity and unpredictable deformations, making it challenging to maintain closed‐loop stability, accurately predict motion trajectories, and implement real‐time control under nonlinear and highly flexible conditions. Additionally, accuracy issues stemming from electromagnetic interference, environmental noise, and sensor drift impede data reliability and spatial resolution in sensing modules.

The future of soft robotics hinges on breakthroughs in materials, actuation, manufacturing, control, and sensing. Self‐powered actuation such as energy harvesting and bioinspired actuator designs are poised to enhance efficiency and adaptability. However, current self‐powered systems remain limited in energy density, long‐term stability, and compatibility with compact designs, especially under varying environmental conditions. Further exploration is needed into hybrid energy harvesting mechanisms, such as simultaneous triboelectric and thermal conversion, to address these limitations. Innovations in self‐healing, shape memory, and multi‐responsive programmable materials, especially those augmented by nanotechnology and advanced composites, promise enhanced durability and functionality in medical, industrial, and environmental applications. High‐resolution multi‐material integration in manufacturing will boost precision, scalability, and structural complexity. Yet challenges persist in aligning disparate material properties, ensuring robust interfacial bonding, and maintaining consistency during large‐scale fabrication. Research is still needed on unified curing strategies, gradient interface design, and real‐time process monitoring to ensure functional integration of soft and rigid materials. Moreover, AI‐driven neuromorphic control strategies will provide real‐time adaptation and intelligent interaction, propelling the evolution of autonomous and responsive soft robotic systems. In sensing, AI‐enabled sensor networks will elevate signal processing, noise filtering, and autonomous decision‐making capabilities. The ultimate vision for soft robotics is seamless integration with the human body or external environments, evolving into an indispensable element of intelligent society. Its future applications extend beyond minimally invasive surgery, precision drug delivery, and rehabilitation therapies to encompass industrial flexible manufacturing, hazardous environment operations, and high‐tech domains like deep‐sea exploration and space missions. Soft robots are also positioned to bring convenience and benefits to everyday life, contributing to smart homes and personalized services.

In conclusion, soft robotics is a leading interdisciplinary technology with great potential for applications from precision medicine to extreme environmental exploration. With ongoing breakthroughs in actuation, materials, manufacturing, control, and sensing, soft robotics is poised to revolutionize multiple industries and offer innovative solutions for sustainable development. Nevertheless, the field still faces fundamental gaps in energy autonomy, long‐term material resilience, and system‐level integration. Future efforts should focus on bridging these gaps through cross‐disciplinary approaches that combine functional material innovation, process engineering, and adaptive intelligence. This will ultimately drive the profound integration of technologies into daily life and foster a smarter and more interconnected future.

## Conflict of Interest

The authors declare no conflict of interest.
